# Identification of miR‐31‐5p, miR‐141‐3p, miR‐200c‐3p, and GLT1 as human liver aging markers sensitive to donor–recipient age‐mismatch in transplants

**DOI:** 10.1111/acel.12549

**Published:** 2016-12-20

**Authors:** Miriam Capri, Fabiola Olivieri, Catia Lanzarini, Daniel Remondini, Vincenzo Borelli, Raffaella Lazzarini, Laura Graciotti, Maria Cristina Albertini, Elena Bellavista, Aurelia Santoro, Fiammetta Biondi, Enrico Tagliafico, Elena Tenedini, Cristina Morsiani, Grazia Pizza, Francesco Vasuri, Antonietta D'Errico, Alessandro Dazzi, Sara Pellegrini, Alessandra Magenta, Marco D'Agostino, Maurizio C. Capogrossi, Matteo Cescon, Maria Rita Rippo, Antonio Domenico Procopio, Claudio Franceschi, Gian Luca Grazi

**Affiliations:** ^1^ DIMES‐ Department of Experimental, Diagnostic and Specialty MedicineAlma Mater StudiorumVia S. Giacomo12BolognaItaly; ^2^ CIG, Interdepartmental Center ‘L. Galvani’Alma Mater StudiorumPzza Porta S. Donato, 1BolognaItaly; ^3^ Department of Clinical and Molecular SciencesUniversità Politecnica delle MarcheVia Tronto 10/AAnconaItaly; ^4^ Center of Clinical Pathology and Innovative TherapyINRCA‐IRCCS National InstituteVia S. Margherita 560124AnconaItaly; ^5^ Department of Physics and Astronomy (DIFA) and INFN Sez. BolognaAlma Mater StudiorumVia Berti Pichat 9/2BolognaItaly; ^6^ Department of Biomolecular SciencesUniversity of Urbino ‘Carlo Bo’UrbinoItaly; ^7^ Center for Genome ResearchLife Sciences DepartmentUniversity of Modena and Reggio EmiliaVia Campi 287ModenaItaly; ^8^ ’F. Addarii’ Institute of Oncology and Transplant Pathology at DIMESS. Orsola‐Malpighi Hospital40138BolognaItaly; ^9^ DIMEC‐Department of General Surgery and Medicine SciencesS. Orsola‐Malpighi Hospital40138BolognaItaly; ^10^ Istituto Dermopatico dell'Immacolata‐IRCCSFLMMVascular Pathology LaboratoryVia dei Monti di Creta 104Rome00167Italy; ^11^ Department of Experimental MedicineSapienzaUniversity of RomeViale Regina Elena 324Rome00161Italy; ^12^ IRCCSInstitute of Neurological Sciences of BolognaBologna40139Italy; ^13^ Istituto Nazionale Tumori ‘Regina Elena’Via Elio Chianesi 53Roma00144Italy

**Keywords:** age‐mismatches, allograft, elderly donors, GLT1, microRNAs, N‐glycans, telomere length

## Abstract

To understand why livers from aged donors are successfully used for transplants, we looked for markers of liver aging in 71 biopsies from donors aged 12–92 years before transplants and in 11 biopsies after transplants with high donor–recipient age‐mismatch. We also assessed liver function in 36 age‐mismatched recipients. The major findings were the following: (i) miR‐31‐5p, miR‐141‐3p, and miR‐200c‐3p increased with age, as assessed by microRNAs (miRs) and mRNA transcript profiling in 12 biopsies and results were validated by RT–qPCR in a total of 58 biopsies; (ii) telomere length measured by qPCR in 45 samples showed a significant age‐dependent shortage; (iii) a bioinformatic approach combining transcriptome and miRs data identified putative miRs targets, the most informative being GLT1, a glutamate transporter expressed in hepatocytes. GLT1 was demonstrated by luciferase assay to be a target of miR‐31‐5p and miR‐200c‐3p, and both its mRNA (RT–qPCR) and protein (immunohistochemistry) significantly decreased with age in liver biopsies and in hepatic centrilobular zone, respectively; (iv) miR‐31‐5p, miR‐141‐3p and miR‐200c‐3p expression was significantly affected by recipient age (older environment) as assessed in eleven cases of donor–recipient extreme age‐mismatch; (v) the analysis of recipients plasma by N‐glycans profiling, capable of assessing liver functions and biological age, showed that liver function recovered after transplants, independently of age‐mismatch, and recipients apparently ‘rejuvenated’ according to their glycomic age. In conclusion, we identified new markers of aging in human liver, their relevance in donor–recipient age‐mismatches in transplantation, and offered positive evidence for the use of organs from old donors.

**Abbreviations**
ALP: alkaline phosphatase
ALT: alanine aminotransferase
ARDD3: arrestin domain containing 3
AST: aspartate aminotransferase
DSA‐FACE: (DNA sequencer‐aided, fluorophore‐assisted carbohydrate electrophoresis)
ELL2: elongation factor, RNA polymerase II, 2
GGT: gamma‐glutamyl transpeptidase
GLT1: (or SLC1A2 or EAAT2) solute carrier family 1 (glial high‐affinity glutamate transporter), member 2
IBIL: indirect bilirubin
IHC: immunohistochemistry
miRs: microRNAs
RT–qPCR: reverse transcriptase–quantitative polymerase chain reaction
TBIL: total bilirubin

## Introduction

The high request of organs moves toward an increased use of marginal donors, including organs from old donors usually transplanted into younger recipients. Within the context of orthotopic liver transplants, clinical evidence suggests that livers from aged donors (≥ 70 years) do have function and duration comparable to those achievable with livers from young donors (Cescon *et al*., 2003, 2008; Gastaca *et al*., 2005; Karpen, 2010; Bertuzzo *et al*., 2016).

The possible explanations of this unexpected finding are the following: (i) the aging rate of the liver is lower with respect to other organs and tissues; (ii) livers from old donors in relatively good clinical conditions can improve their function when transplanted into young recipients. These two possibilities are not mutually exclusive. Regarding the first possibility, it has been reported that hepatocytes can restore up to 75% of a surgically removed or damaged liver. Studies showed that liver gradually declines in volume, drug metabolism, and detoxification activity during aging. Data from literature, based on studies in old rodents and elderly humans, indicate an age‐related decline of the liver due to reduced regenerative capacity and decreased gene expression (Sersté & Bourgeois, 2006; Gagliano *et al*., 2007; Schmucker & Sanchez, 2011), even if the liver appears to be relatively protected in comparison with other organs from marked age‐dependent changes.

Concerning the second possibility, no data are available in humans, despite the growing evidence that the young milieu can rejuvenate brain, muscle, and liver of old mice (Conboy *et al*., 2005; Villeda *et al*., 2014).

We addressed these points by studying liver biopsies from organ donors of different ages and by analyzing the cases with the largest age‐mismatches between donors and recipients of the same gender. Further, we tested also plasma from recipients, not only before and after transplant, but also stratifying transplanted recipients on the bases of donor age‐mismatches. Two different types of markers, that is, liver microRNAs (miRs) and plasma N‐glycans, were specifically taken into account. The former is because of its high relevance in controlling gene expression and molecular pathways also during aging (Chen & Meister, 2005; Olivieri *et al*., 2013) and since at present no data are available regarding age‐related miRs expression changes in human liver. The latter is because of their recognized role in the detection of liver function and biological age (Dall'Olio *et al*., 2013; Krištić *et al*., 2014). Noteworthy, recent data indicate the role of miRs in the regulation of specific glycan biosynthetic enzymes, thus suggesting a tight connection among these markers (Bernardi *et al*., 2013).

Glycosylation is a frequent co/posttranslational modification of proteins, which modulates a variety of biological functions. Plasma or serum N‐glycomics, that is, the comprehensive analysis of N‐linked oligosaccharides attached to asparagine residues of glycoproteins (also known as N‐glycans), has been demonstrated to be an useful tool to study N‐glycosylation changes in chronic liver diseases (Blomme *et al*., 2009) and aging (Vanhooren *et al*., 2007) because a part of glycosylated serum proteins are synthesized and secreted by the liver. Accordingly, glycomic markers have been developed to test different liver pathologies, such as liver fibrosis progression (named ‘GlycoFibro test’) (Vanderschaeghe *et al*., 2009), cirrhosis (named ‘GlycoCirrho test’) (Callewaert *et al*., 2004), nonalcoholic steatohepatitis (named ‘GlycoNash test’) (Chen *et al*., 2009), and biological age (named ‘GlycoAge test’) (Vanhooren *et al*., 2007, 2008, 2010).

The objectives of our study were essentially two: (i) to study the aging of human liver (age range: 12–92 years) by focusing on miRs and their mRNA targets and proteins as candidate markers, taking telomere length as a reference marker of senescence and (ii) to evaluate the effects of donor–recipient age‐mismatch by analyzing miRs expression in transplanted organs and by performing the Glycotests in recipient plasma taking into account consistent donor–recipient age‐mismatches. Glycotests were also correlated to standard hemato‐biochemical analysis of liver function assessment.

## Results

Results are presented in two sections: liver aging and donor–recipient age‐mismatch effects in human transplant.

### Liver aging

#### MiRs profiling

**Figure 1:**
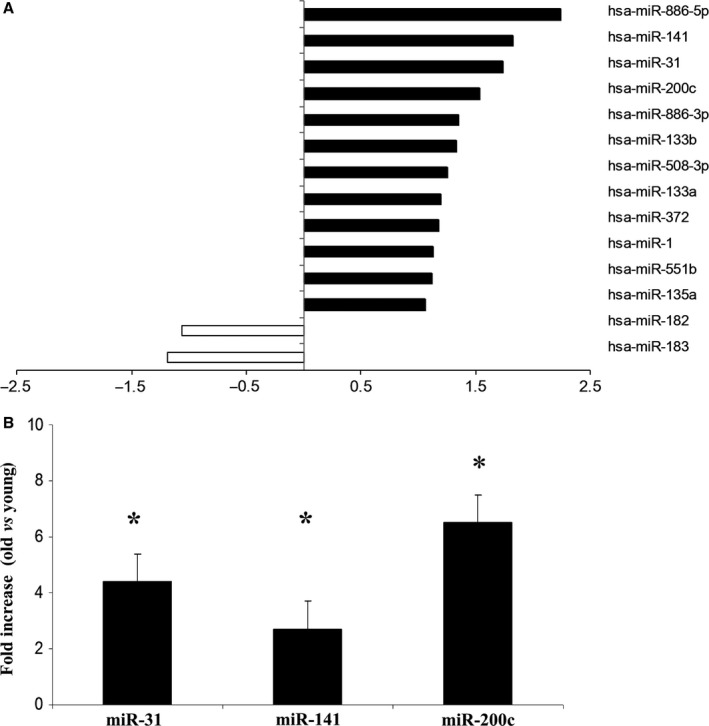
(A) Age‐related miR profiling results. The most significant results analyzing liver biopsies obtained from 12 men (three old > 70 years vs. nine younger donors). Each bar corresponds to expression fold changes, using ΔCt and ΔΔCt methods with normalization on the median value of miRs expression in each card (see material methods). A 1.5‐fold change or greater was considered significant. (B) MiRs relative expression validation in liver from 26 male donors. miR relative expression by RT–qPCR **i**n 26 liver biopsies (nine old > 70 years vs. 17 younger donors). Data were normalized against RNU44 expression levels. *P values <0.05, independent samples t‐test.

To assess miRs expression, liver specimens obtained from 12 male subjects of different age (13–87 years) were screened using miR cards (card A), containing the most known miRs. MiRs expressed at detectable level in more than 80% of samples were considered and, using these filtering criteria, 290 miRs of the 365 analyzed were included in the final analysis. Overall, the most expressed miRs include those known to be liver specific, such as miR‐122, which was confirmed to be among the most expressed (Fig. S1). When the age of the donor was considered, 4 of the 290 miRs, i.e., miR‐31‐5p, miR‐141‐3p, miR‐200c‐3p, and miR‐886‐5p, showed an increase (a fold change higher than 1.5‐fold) in ≥ 70‐year‐old donors with respect to younger age donors (Fig. 1, panel A). We did not identify any miRs significantly decreased with age.

#### Validation of miR results

The top‐ranking miRs hyperexpressed in old vs. young donors (miR‐31‐5p, miR‐141‐3p, and miR‐200c‐3p and miR‐886‐5p) were selected to be confirmed *via* RT–qPCR. Data were validated in the same samples used for profiling and in additional 14 biopsies from male donors of different age. The results obtained in a total of 26 liver biopsies (*n*. 17 < 70 years; *n*. 9 ≥ 70 years) are reported in Fig. 1, panel B. RT–qPCR analysis confirmed the results obtained by card profiling for three of four miRs, i.e., miR‐31‐5p, miR‐141‐3p, and miR‐200c‐5p. In particular, expression levels were significantly higher in old (≥ 70 years) compared to younger (< 70 years) donors regarding miR‐31‐5p (4.5‐fold, *P* = 0.007), miR‐141‐3p (2.7‐fold, *P* = 0.034), and miR‐200c‐3p (6.7‐fold, *P* = 0.017), while miR‐886‐5p was not confirmed to be significantly changed.

**Figure 2:**
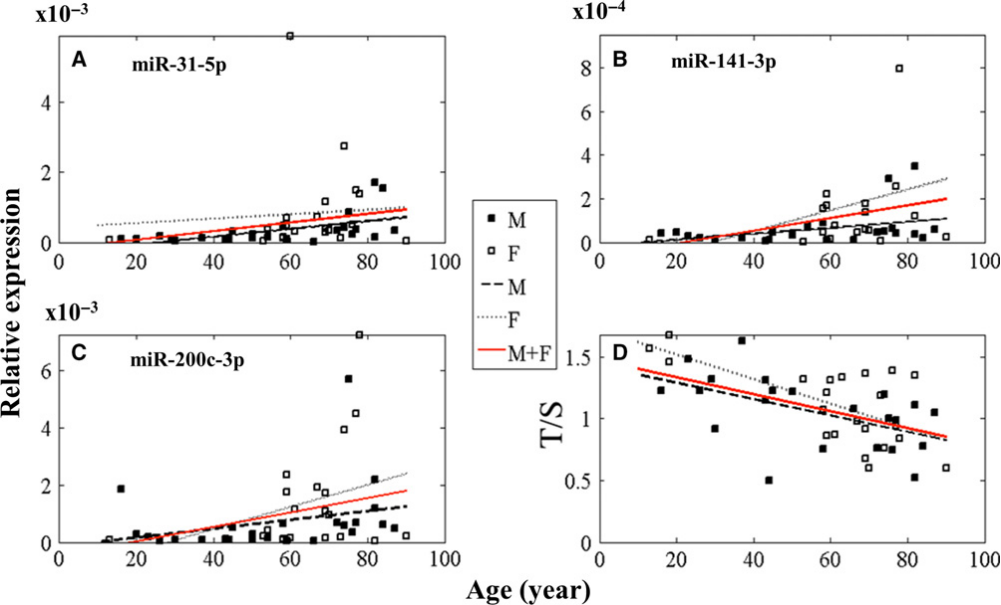
MiR‐31‐5p (panel A), miR‐141‐3p (panel B), miR‐200c‐3p (panel C) relative expression and telomere length (T/S, panel D) in 45 liver biopsies from 13 years up to 90 years donors vs. age. Open squares: Females (*n* 19); closed squares: males (*n* 26). Red line: linear regression over all samples; dotted line: linear regression over female samples; dashed line: linear regression over male samples. Spearman's correlation coefficient r between age and each parameter (miRs, T/S) is calculated over all samples. Panel A: miR‐31‐5p *r* = 0.3703, *P* = 0.0221; panel B: miR‐141‐3p *r* = 0.3336, *P* = 0.0407; panel C: miR‐200c‐3p *r* = 0.3116, *P* = 0.0568; panel D: telomere length *r* = −0.4625, *P* = 0.0035

The miR‐31‐5p, miR‐141‐3p, miR‐200c‐3p found to be significantly overexpressed and RT–qPCR‐validated in livers from old male donors and were further studied (RT–qPCR) in liver samples from 19 female donors of different age. In Fig. 2 (panels A–C), the expression of miR‐31‐5p, miR‐141‐3p, miR‐200c‐3p in livers from all donors (26 male and 19 female ranging from 13 up to 90 years) is reported. The correlation of miRs expression with age was confirmed in males, while in females, no significant correlation emerged, even if an age‐related trend similar to that observed in males was found. Considering male and female samples together, data confirmed a significant age‐related correlation of miR‐31‐5p, miR‐141‐3p, and weakly for miR‐200c‐5p (*P* = 0.05) expression with age (Table S1A). Moreover, the age‐related profiles of the three miRs were significantly correlated one another (Table S1B).

#### Telomere length

A significant age‐related telomere length shortening was observed in the same 45 biopsies analyzed for miR expression (Fig. 2, panel D). This shortening is confirmed to be significant in male donors (Table S1A). However, no significant correlation was found between telomere length and miR‐31‐5p, miR‐141‐3p, miR‐200c‐3p expression levels (Table S1B).

#### mRNA expression patterns

To identify the possible mRNA targets of the age‐related upregulated miRs, we performed a global liver gene expression profiling. Seventy‐six mRNA probes were significantly downregulated in the livers of elderly subjects (log ratio < −0.4, *P* < 0.05), as listed in Table S2A–B.

#### Association of miRs and their predicted targets

mRNAs that are predicted to be the direct targets of specific upregulated miRs should be in general expressed at lower levels. Predicted targets were identified using SID1.0 program (Albertini *et al*., 2011), and they were coupled with the transcriptomic data. Among the 76 downregulated mRNA in livers from elderly subjects, only three were reported to have conserved 3′UTR sites targeted from the selected miRs as follows: (i) solute carrier family 1‐glial high‐affinity glutamate transporter member 2 (GLT1 or SLC1A2); (ii) elongation factor, RNA polymerase II, 2 (ELL2); and (iii) arrestin domain containing 3 (ARDD3).

#### Validation of GLT1, ELL2, and ARDD3 gene transcripts

**Figure 3:**
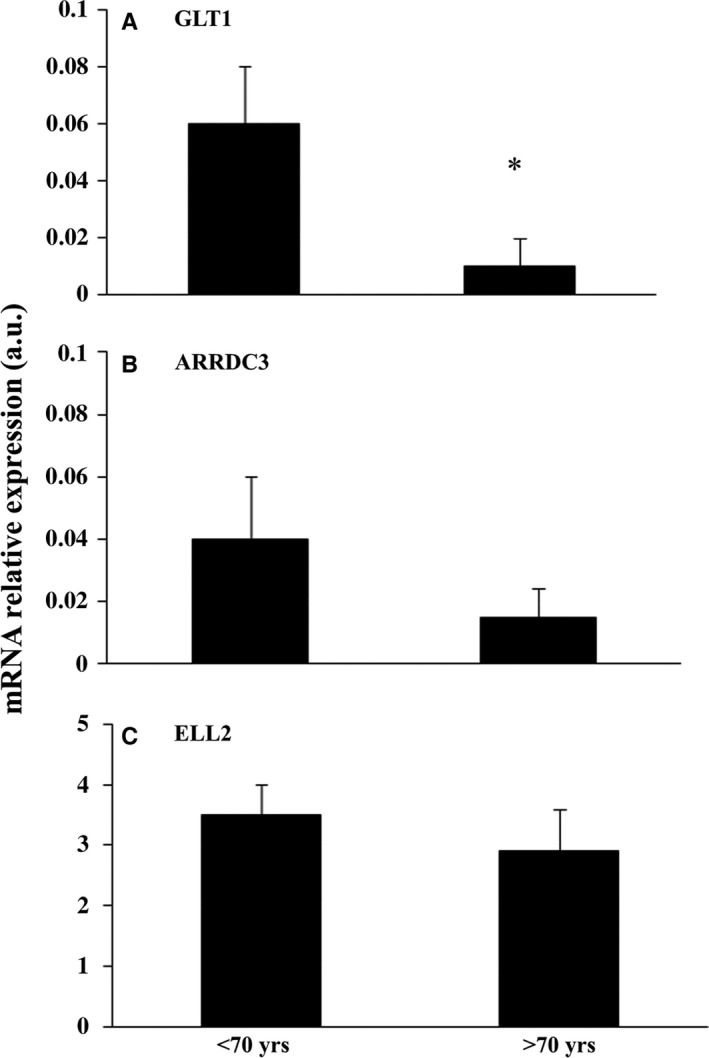
mRNA expression of GLT1 (SLC1A2, panel A), ARRDC3 (panel B), and ELL2 (panel C) in 26 liver biopsies. RT–qPCR expression analyses of mRNA target levels in liver of young (*n* 14, < 70 years) and old (*n* 12, ≥ 70 years) donors. Data were normalized against GAPDH expression levels and reported as the mean value ± SD. *P values <0.05, independent samples t‐test.

The three transcripts found downregulated by means of Affymetrix chips were validated via RT–qPCR in liver male specimens from 14 young (< 70 years) compared to 12 old (≥ 70 years) donors. A decrease expression of the three mRNA targets was observed in livers from old vs. young donors, but significance was reached only in the case of GLT1 (or SLC1A2) (Fig. 3, panels A–C).

#### Immunohistochemistry (IHC)

**Figure 4:**
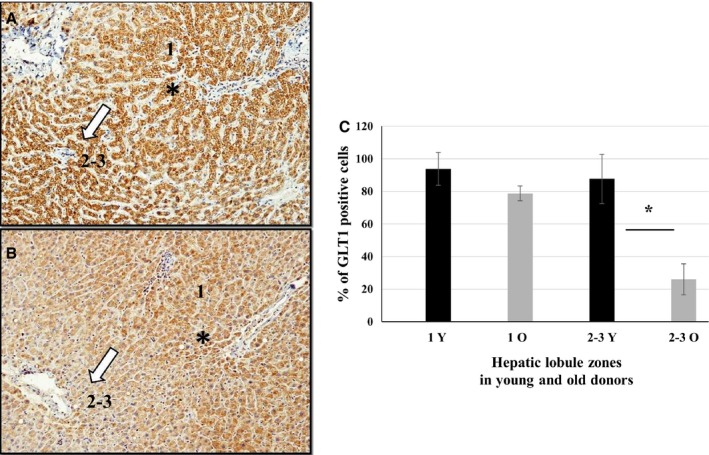
GLT1 immunohistochemistry analysis of young (Y, panel A) and old (O, panel B) donors. A representative analysis is reported in the panels A and B. Perivenular zones (asterisk, Z‐1) and periportal/centrilobular zones (open arrow, Z‐2‐3) were analyzed in seven young and seven old **(≥ **70 years) donors. Data are reported as mean ± SD in panel C: 257 (150 from Y; 107 from O) perivenular zones (Z‐1) and 198 periportal/centrilobular zones (Z‐2‐3) (108 from Y, 90 from O) were scored for GLT1 staining (two grades of intensity were relieved, i.e., weak and strong). *P values <0.05, nonparametric Mann–Whitney U‐test.

A total of 257 periportal and 198 centrilobular zones were counted in paraffin‐embedded liver biopsies from seven young and seven old donors (> 70 years). One hundred and fifty periportal zones were examined in samples from young subjects, and a mean of 93.8% (±10.1) showed strong GLT1 immunoreactivity. Similarly, 107 periportal zones were examined in biopsies from old subjects and a mean of 78.2% (±4.6) showed strong GLT1 positivity without varying significantly between the two age groups. As far as centrilobular zones are concerned, 108 were counted in samples from young subjects, and a mean of 87.7% (±15.2) were strongly positive. In the 90 centrilobular zones examined from old donor samples, only 26.5% (±9.6) centrilobular zones showed the same strong reactivity. Therefore, GLT1 expression in centrilobular zones was significantly lower in older subjects (*P* = 0.024), as reported in Fig. 4 (panels A–C). Furthermore, the histological features characterizing liver specimens from young and old donors were different, as expected. These morphological findings were in accordance with the commonest age‐dependent features described in the literature (Odze & Goldblum, 2009), and results are shown in Fig. S2 (panels A, B).

### GLT1 (SLC1A2) is a direct target of miR‐31‐5p and miR‐200c‐3p

**Figure 5:**
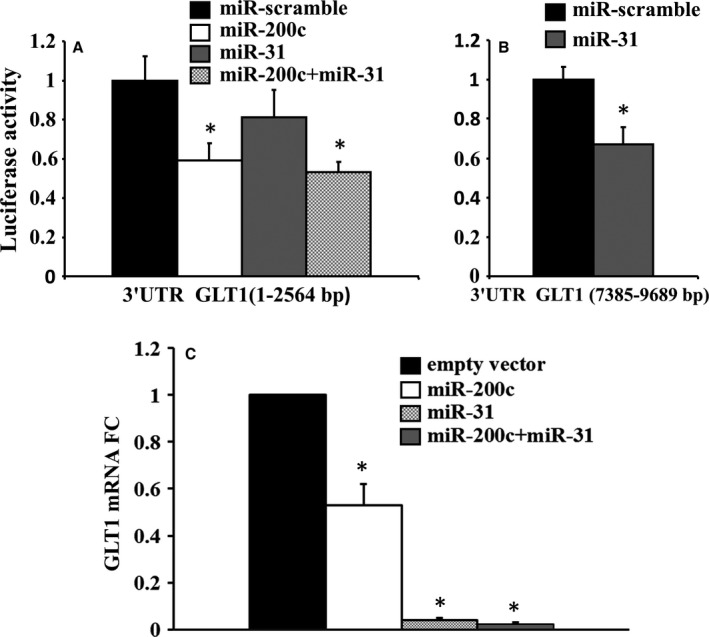
Luciferase (panels A, B) and functional assays (panel C). HEK 293 was transfected with firefly luciferase constructs containing different portions indicated in figure of 3′UTR of GLT1 (SLC1A2) gene. A. Each 3′UTR cloned in pEZX‐MT06 plasmid was cotransfected with a plasmid encoding either miR‐200c or miR‐31 or cotransfected with both miRs. As control, miR‐scramble sequence was used. Values were normalized according to renilla luciferase activity (*n* = 3 in triplicate; *P < 0.02). miR‐200c downmodulated the luciferase activity of 3′UTR construct. B. The different 3′UTR cloned in pEZX‐MT06 plasmid was cotransfected with a plasmid encoding either miR‐200c or miR‐31 or miR‐scramble. miR‐31 downmodulated the luciferase activity of 3′UTR construct (*n* = 3 in triplicate; *P < 0.02). C. A significant decrease in GLT1 mRNA expression was found in HepG2 cells transfected with miR‐31 mimic, miR‐200c mimic, and miR‐31 plus miR‐200c mimics (*n* = 3; *P < 0.05).

GLT1 3′UTR is 9689 bp long and contains different miR‐31‐5p and miR‐200c‐3p seed sequences. In particular, it contains four binding sites for miR‐200c‐3p, one broadly conserved among species at position 1806‐1813 nt of GLT1 3′UTR and other three seed sequences not conserved among species (at positions 1465‐1471nt, 6831‐6837nt, 7086‐7092nt) (Fig. S3 panels A, B). In addition, miR‐31 has four seed sequences within the 3′UTR of GLT1 gene. The two conserved sites are at positions 277‐284nt and 7559‐7566nt of the 3′UTR; the poorly conserved sites are at 2806‐2812nt and 3183‐3189nt of 3′UTR (Fig. S3 panels A, B). To analyze all these potential miRs binding sites, we used four different 3′UTR luciferase constructs, each containing about 2500 bp of the GLT1 3′UTR partially overlapping among them in order to cover the entire 3′UTR, cloned downstream of a luciferase open‐reading frame. Luciferase activity of the construct containing the sequence 1–2564 bp of the 3′UTR of GLT1 (with two miR‐200c seed sequences, one conserved and one poorly conserved across species, and one miR‐31 conserved sequence) was significantly downmodulated by miR‐200c compared to a miR‐scramble used as control. MiR‐31 was also able to decrease the luciferase activity, although not significantly, and luciferase activity was also downregulated by cotransfection of both miRs, but this decrease is similar to the one obtained by miR‐200c alone (Fig. 5, panel A). Luciferase construct containing the sequence 7385–9689 bp of the 3′UTR, that has the other miR‐31 conserved sequence, was efficiently downmodulated by miR‐31, when compared to the miR‐scramble control (Fig. 5, panel B). Luciferase activity of the other two constructs containing the miR‐31 and miR‐200c seed sequences not conserved among species is not inhibited by miR‐31 or miR‐200c, respectively, when compared to miR‐scramble (Fig. S4 panels A, B). Taken together, these results showed that GLT1 is a direct target of both miR‐200c and miR‐31. Further, functional assays were performed on HepG2 cells, transfected with mimic expression plasmids, that is, miR‐31‐5p and miR‐200c‐3p, and a combination of them (miR‐31 + miR‐200c), using an empty vector as control. After 72 h of transfection, the expression of miR‐200c was increased in cells transfected with miR‐200c mimic plasmid, either alone or associated with miR‐31 mimic plasmid. When HepG2 cells were transfected with miR‐31, either alone or plus miR‐200c mimic plasmids, the expression level of miR‐31 was significantly higher when compared with empty vector transfected cells (Fig. S5, panels A, B). A significant decrease of GLT1 mRNA expression level was observed with transfection of miR‐31‐5p and miR‐200c‐3p alone and in combination. However, the highest inhibition of GLT1 mRNA expression level was observed with miR‐31 transfection, alone or in combination, as shown in Fig. 5, panel C.

### Donor/Recipient age‐mismatches

#### Effects of donor/recipient age‐mismatches on miR‐31‐5p, miR‐141‐3p, miR‐200c‐3p expressions in transplanted liver

**Figure 6:**
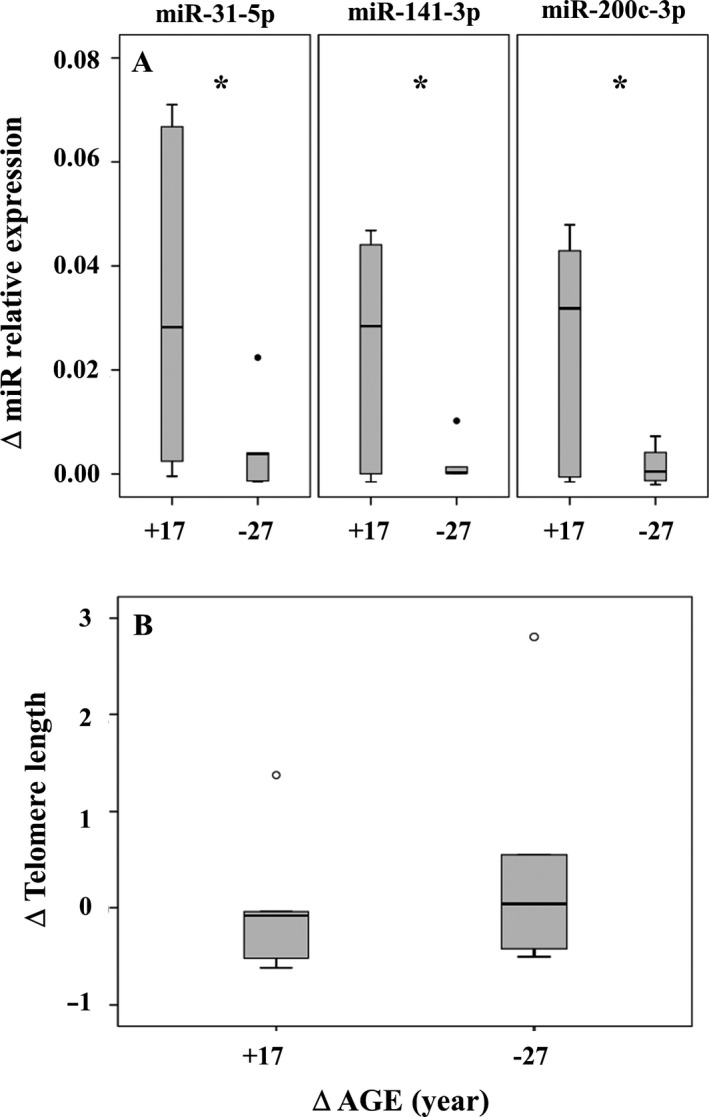
Δ miRs relative expression (panel A) and telomere length (panel B) in liver before (donor) and after (recipient) transplant. A. MiR‐31‐5p; miR‐141‐3p; miR‐200c‐3p were evaluated in 11 recipients: six older than their donors (Δ age average: +17 years); five younger than their donors (Δ age average: −27 years) after 15 ± 7 months and 10 ± 2 months from transplant, respectively. B. Telomere lengths were evaluated in the same donor–recipients (except one). *miR‐31‐5p; miR‐141‐3p; miR‐200c‐3p: *P* values = 0.05; 0.03; 0.02, respectively (one‐side paired samples t‐test).

To evaluate the possible effect of the recipient environment on miR‐31‐5p, miR‐141‐3p, miR‐200c‐3p expressions, we took advantage of 11 cases where the donor/recipient age‐mismatch was consistent (from −27 to + 17 years). Accordingly, miR‐31‐5p, miR‐141‐3p, and miR‐200c‐3p were assessed in six recipients (age: 60 ± 8 years) older than their donors (age: 42 ± 12 years) and in five recipients (age: 49 ± 14 years) younger than their donors (age: 72 ± 6 years) at 15 ± 7 months and 10 ± 2 months after transplantation, respectively. The results are reported in Fig. 6, panel A. A significant increase (*P* ≤ 0.05) of Δ (difference between donor and recipient) expression of all miRs was observed in livers exposed to an aged environment at variance with livers exposed to a younger environment. No change was observed regarding Δ telomere length (from −27 to + 17 years) in the transplanted organs of the same age‐mismatched cases (Fig. 6, panel B).

#### Glycotests in recipient plasma

**Figure 7:**
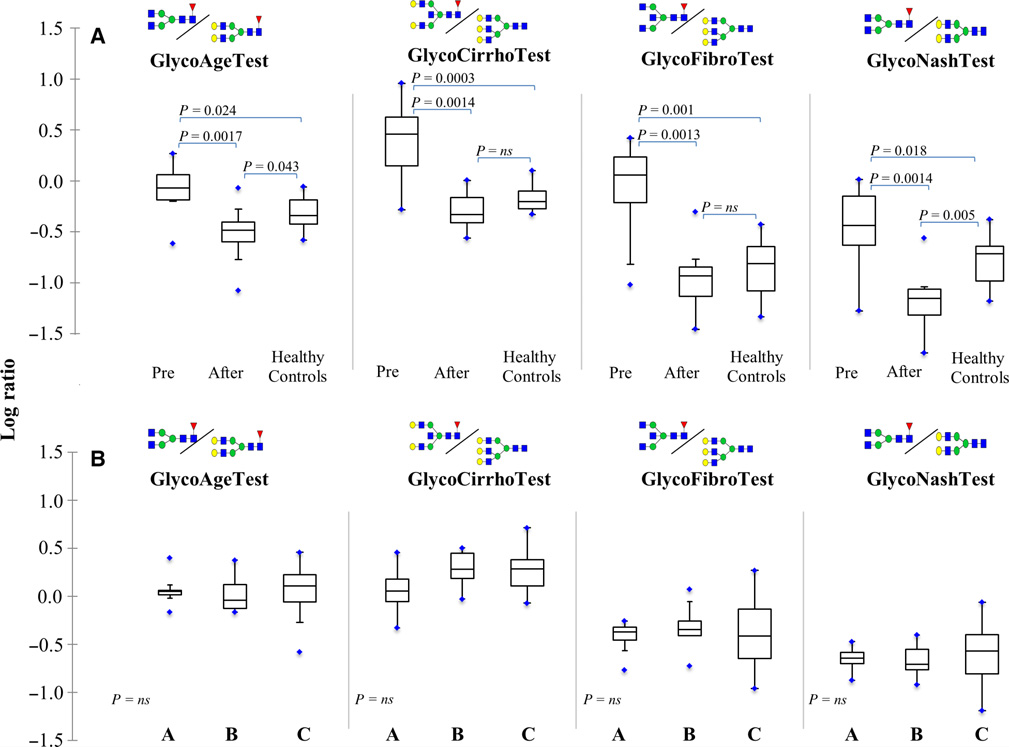
Plasma Glycotests. A. Glycotest values of 14 recipients before and after liver transplant and of ten age‐matched healthy volunteers. The box‐plots show median, minimal and maximal values (whiskers), mean (red cross) interquartile ranges (box), as well as outliers (blue circles) for the log ratio of relative intensity of the N‐glycan features listed over the plots referring to each Glycotest. Differences before and after transplantation were tested by Wilcoxon signed‐ranks differences post‐transplantation, and healthy volunteers were tested by Kruskal–Wallis. For each comparison, the *P* ‐values are indicated. B. Glycotest values of recipients after transplant, stratified in three groups (A, B, C) according to the age‐mismatches between donors and recipients: A. Donors younger (31 ± 15.6 years) than recipients (54.9 ± 8.3 years; *n* = 9) with age‐mismatch range from −42 to −14 years; B. Donors and recipients with similar ages (*n* = 9), age‐mismatch range from −7 to +8 years; C. Donors older (76.3 ± 9.3 years) than recipients (46.4 ± 10 years; *n* = 18) with age‐mismatch range from +53 to +12 years. Differences among the groups were tested using Kruskal–Wallis.

Profiles of plasma N‐glycans from a representative recipient before and after transplant together with a healthy age‐matched control are shown in Fig. S6. Glycotests, before and after transplant (14 cases), are shown in Fig. 7, panel A, where GlycoAge test is the log ratio of NGA2F/NA2F; GlycoCirrho test is log ratio of NA2FB/NA3; GlycoFibro test is log ratio of NGA2FB/NA3; and GlycoNash test is log ratio of NGA2F/NA2. All the Glycotests resulted decreased after transplantation showing similar values to healthy and age‐matched volunteers (not transplanted), in particular GlycoAge test was significantly lower in transplanted than not transplanted and healthy subjects. Furthermore, alkaline phosphatase (ALP), gamma‐glutamyl transpeptidase (GGT), albumin, aminotransferases (ALT, AST), total bilirubin (TBIL), and indirect bilirubin (IBIL) were evaluated before and after transplant when feasible (Table S3), and only TBIL and IBIL resulted significantly changed. Glycotests were performed on 36 plasma samples obtained from recipients after transplant and stratified according to age‐mismatch between donors and recipients. In Fig. 7 (panel B): group A consists of donors younger than recipients (*n* = 9) with age‐mismatch ranging from −42 to −14 years; group B consists of donors and recipients with similar ages (*n* = 9) and age ranging from −7 to +8 years; group C comprises donors older than recipients (*n* = 18) with age‐mismatch ranging from +53 to +12 years. No significant effect of age‐mismatch was evident by comparing the three groups. An increase in variance was particularly evident in the group C, but it was not significant. In addition, Glycotests were correlated with ALT, AST, TBIL, and IBIL (using data available for all the 36 recipients after transplant) and results indicated that GlycoAge, GlycoFibro, and GlycoNash tests strongly correlated with TBIL and IBIL, the most relevant markers of liver function, as reported in the correlation map (Fig. S7).

## Discussion

This study aimed at collecting biological data to explain why livers from old donors may be successfully used for transplants. Moreover, we addressed the biological effect of age‐mismatch between donor and recipient, a topic rather neglected despite its great potential clinical interest. Aging liver was assessed by miRs and mRNA expressions in a cross‐sectional design, and recipient status was evaluated by new systemic glycomic markers capable of assessing hepatic functionality by means of different Glycotests (Callewaert *et al*., 2004; Vanhooren *et al*., 2007, 2008, 2010; Chen *et al*., 2009; Vanderschaeghe *et al*., 2009) within a time series design. Inherent in these studies are limitations regarding the low number of biopsies from females (gender distribution cannot be *a priori* decided), the small quantity of needle biopsies after transplants, and the clinical heterogeneity of the recipients and their immunosuppressant protocols. Further, one possible limitation is that some of the observed changes can be due to age‐dependent variations in cell composition.

The search for aging‐markers identified three of 295 detectable miRs, i.e., miR‐31‐5p, miR‐141‐3p and miR‐200c‐3p, as significantly increased in livers from old (> 70 years) vs. younger donors. Notably, in our study, miR expression started to increase after 60–70 years of age and no miR resulted decreased, confirming data previously reported on the liver aging in mice (Maes *et al*., 2008; Bates *et al*., 2010) and rats (Li *et al*., 2011).

MiR‐31 and miR‐200 family expressions were reported to be increased in human liver diseases such as nonalcoholic‐related steatohepatitis‐nonalcoholic fatty liver disease (NASH‐ NAFLD), and miR‐141 was found modified in patients with HCV infection, as recently reviewed (Lakner *et al*., 2011). In the present work, all the explanted livers were implanted and none of them was discarded as nonsuitable. In fact, they did not reveal any types of NASH‐NAFLD and/or active HCV infection as assessed by histological evaluation. Despite being free of such specific disease signs, morphological data confirmed that the livers did not escape the aging process.

MiR‐200c, belonging to the same family of miR‐141, was reported to be upregulated in endothelial cells where apoptosis and cell senescence have been induced by oxidative stress (Magenta *et al*., 2011). A similar role of miR‐141 in cell senescence was recently reported in human diploid fibroblasts (Dimri *et al*., 2013). As a further marker of senescence, we measured telomere length, which showed an age‐related shortening. A recent paper reported a shortening of telomeres with age in human liver and the authors also showed that cells responsible for telomere attrition were not hepatocytes and cholangiocytes, but sinusoidal and stellate cells (Verma *et al*., 2012). When we checked a relationship between telomere shortening and miR‐31‐5p, miR‐141‐3p, miR‐200c‐3p increase, no correlation was found, suggesting either that different molecular mechanisms are responsible for these two types of age‐related markers or that they refer to different cell types. Another possible confounding factor is gender. Verma *et al*. considered males and females all together, while we found a stronger correlation between age and telomere shortening in male than in female samples, but the relatively low number of young female samples in our collection likely biases this observation.

MiR‐141 and MiR‐200c have recently been reported to directly regulate an important enzyme, that is, alpha‐L‐fucosidase 2 (FUCA2), involved in the fucosylation of N‐glycans modifications (Agrawal *et al*., 2014), but no change of its transcript levels was found by GeneChip^®^ system in the liver samples we studied. To this regard, gene expression profiling revealed that some of mRNAs, putatively targeted by these miRs, a downregulation in aged liver, and GLT1, ELL2, and ARDD3 emerged as the leading significant mRNAs. In particular, GLT1 was among the top‐ranking transcripts showing the most significant change with age. Validation experiments confirmed a decrease in the three mRNAs in aged livers, but only GLT1, a key regulator of glutamine metabolism, reached statistical significance. GLT1 3′UTR shows four putative target sites for miR‐31‐5p and four putative target sites for miR‐200c‐3p, suggesting that it can be targeted by both miRs. Here, we demonstrate for first time that GLT1 is a direct target of both miR‐31‐5p and miR‐200c‐3p and is functionally downregulated by these two miRs. As far as GLT1 protein level is concerned, we showed a decreased expression pattern in livers from old compared with young donors in specific hepatic areas, such as periportal/centrilobular zones, suggesting a possible functional role played by deregulated miRs, and revealing for the first time an age‐related remodeling architecture of GLT1 expression in human hepatic lobules. In an *in vitro* system, GLT1 protein decrease was not revealed (data not shown), likely because the transient transfection of selected miRs for 72 h in HepG2 is not sufficient to induce a significant modification of GLT1 protein expression, likely depending on the specific protein turnover and on tissue‐specific regulation of this protein. In fact, GLT‐1 membrane protein is strongly expressed by perivenous hepatocytes of rat liver (Berger & Hediger, 2006) due to a metabolic zone architecture, that appears maintained by Dicer 1, a key regulator of miR biogenesis (Sekine *et al*., 2009), in accord with our observation in humans showing, for the first time, an age‐associated GLT1 remodeling. As a possible role of GLT1 is to support glutamine synthesis, our results suggest a major role for genes involved in glutamine pathway in the aging of human liver. These results are in accordance with those recently obtained in animal models (mice and rats), where a transcriptional remodeling during liver aging, related to the glutamine pathway, such as glutamate cysteine ligase (Gclc) (Shenvi *et al*., 2012) and other metabolites of the polyamine biosynthesis (Maes *et al*., 2008; Kwekel *et al*., 2010) was demonstrated.

Further, taking advantage of a small number of consistent (from −27 years up to +17 years) donor–recipient age‐mismatches, we checked whether miR‐31‐5p, miR‐141‐3p and miR‐200c‐3p maintained the expression level of the donor in transplanted liver or whether it was influenced by the age of the recipient. On the whole, we report that miR‐31‐5p, miR‐141‐3p, and miR‐200c‐3p expressions significantly increased in old recipients, suggesting that these miRs can be assumed as a potential liver sensors of old environment. It is at present unclear to what extent this phenomenon is related to an accelerated aging of the transplanted organ. On the opposite site of the age‐mismatch, recipients younger than donors significantly kept miR level similar to previous environment (donor), thus apparently avoiding the increase in miR‐31‐5p, miR‐141‐3p, and miR‐200c‐3p expressions, found in recipients older than donors. Due to the low amount of biological material (needle biopsies), it was not possible to assess GLT1 mRNA.

‘Rejuvenation’ effects have been reported in ‘heterochronic parabiosis’ experiments in mice (Conboy *et al*., 2005; Villeda *et al*., 2014), while in humans, the possibility to study rejuvenation effects are challenging. Liver transplantation, when a consistent donor–recipient mismatch is present, may be considered a sort of parabiotic experiments, thus representing a good model to study the effect of systemic environment (recipient) on the transplanted organ. We found an impressive effect of recipients, but further studies on a higher number of donor–recipient age‐mismatches need to confirm and extend the results here reported. Other important variables in liver transplants are viral infections. Three of eleven recipients were HCV positive and one HBV positive, but this number was too small to evaluate viral effects on miRs expression. Interestingly, data from literature suggest that depletion of miR‐141‐3p inhibited HCV replication in an *in vitro* system (Banaudha *et al*., 2011). We can argue that the lack of miR‐141‐3p increase in younger HCV+ recipients might contribute to their graft success.

Finally, we used an innovative glycomic approach to assess recipient biological age (GlycoAge test) as well as liver function (hepatic‐Glycotests, namely GlycoCirrho test; GlycoFibro test; and GlycoNash test, known to be highly sensitive) after transplant. Accordingly, we were able to show for the first time that recipient biological age appears to be extremely high before transplant (similar to extreme old ages, see Vanhooren *et al*., 2010), becoming after transplant more similar, or even younger, than healthy age‐matched volunteers. At present, an effect of immunosuppressant therapy on galactosylation of different plasma proteins cannot be excluded. In addition, glycohepatic tests showed improvement of liver function after transplantation and were highly correlated with the most important marker of liver function, that is, total and indirect bilirubin. Importantly, by stratifying Glycotests results on donor–recipient age‐mismatches, no significant differences were found. These data point to additional evidence showing that livers from old donors are functionally similar to those from young subjects, as recently suggested by clinical observations (Thorsen *et al*., 2015).

On the whole, miR‐31‐5p, miR‐141‐3p, and miR‐200c‐3p have been identified as markers of liver aging whose expression is sensitive to an ‘old environment’ (recipients older than donors). Moreover, we identified GLT1 as a target of two of the above‐mentioned miRs (miR‐31‐5p; miR‐200c‐3p) and a new marker of human liver aging. The markers we identified also suggest that the liver starts aging quite late as miR expression increase starts around 60–70 years of age, according to our cross‐sectional study. These results should be validated in larger cohorts and possibly in longitudinal studies, which at present appear difficult to perform. In any case, liver function appears well preserved after transplant, even when livers from old donors are used, further supporting the use of aged ‘marginal donors’ in liver transplantation.

## Experimental Procedures

### Donors

This study, in the context of the protocol of donor–recipient allograft performed at General Surgery and Transplant Unit (S. Orsola‐Malpighi Hospital, Bologna, Italy), received the approval of the local ethical committee (code: 44/2008/Tess). Seventy‐one liver biopsies of about 50‐60 mg were obtained from 71 heart‐beating and brain death donors aged from 12 up to 92 years. Tissue samples were obtained just after midline incision and thus before aorta cross‐clamping and flushing. All liver grafts were used for liver transplantation, and none of them was discarded as nonsuitable.

Death causes of each donors are described in supplementary material (donors and biopsies file). Hepatic available indicators, that is, bilirubin, ALT, AST, and GGT collected from all donors, were within normal ranges in all cases, and looking at the differences between the two young (< 70 years) and old (≥ 70 years) groups, we did not found significant changes (data not shown). On the other hand, the judgment of suitability of the liver grafts for transplant was based on the macroscopic aspect (as assessed by the surgeon in charge for organ retrieval) and on histology report, according with standardized procedure (Ishak *et al*., 1995; Ravaioli *et al*., 2009). No donor organs were obtained from executed prisoners or other institutionalized persons.

### Recipients

All recipients (see supplementary material, recipient and blood sample file) had severe liver pathologies often due to HCV or HBV infections, such as hepatocarcinoma, hepatitis, and cirrhosis among others. Underlying liver diseases indicating liver transplantation were HCV‐related cirrhosis in 14 of 36 (38.9%) cases (eight with HCC), HBV‐related cirrhosis in eight of 36 (22.2%) (four with HCC), alcoholic cirrhosis in seven of 36 (19.4%) (three with HCC), polycystic disease in two of 36 (5.5%), while cryptogenic cirrhosis, primary biliary cirrhosis, primary sclerosing cholangitis, Budd–Chiari syndrome, and amyloidosis were in one of 36 (2.8%) for each mentioned diseases.

After transplant (6–23 months), needle biopsies were obtained from 12 recipients (from one of them, RNA/DNA was not sufficient for analysis) being necessary for those patients. All the specimens were immediately frozen in liquid nitrogen at both the liver graft retrieval and post‐transplant follow‐up times. Thirty‐six plasma samples were collected from 36 recipients (only 14 samples were available for two time points: before and after the transplant; see recipient and blood sample file in supplemental material). All the recipients were in good clinical condition early after transplant and at the time of follow‐up evaluations. Later on, 7 of 36 (19.4%) developed histology‐proven hepatitis C recurrence, with or without alteration of biochemical liver function tests. In addition, five patients (13.9%) experienced one acute rejection episode, which was successfully treated with immunosuppression modulation before obtaining the study follow‐up biopsies in all cases. All of them were treated with immunosuppressant drugs (maintenance: 55.6% based on tacrolimus, 8.3% based on cyclosporine, and 36.1% *ad hoc* combination of drugs). In supplemental material, drug treatments were reported for each recipient both at induction and at maintenance phases.

### Experimental design

The experimental design was initially set up for the study of liver aging. MiRs profiling was performed on two groups of young and older male donors (> 70 years). On the whole, miRs were validated in 45 liver biopsies from male and female donors of different ages (range: 13–90 years) and telomere length was measured on the same samples. mRNA transcriptome (using 12 different biopsies) was performed by GeneChip^®^ and mRNA targets were validated in 26 samples (17 young vs. nine old donors). Additional 14 biopsies were used for immunohistochemical investigations (seven young vs. seven old subjects). Further, 11 cases of donor‐recipient age‐mismatches (Δ age: from −27 to +17 years) were used to assess miRs expression in biopsies before and after transplant. On the whole, 71 biopsies were used, as described in supplementary material (donor and biopsy file).

As far as recipient samples are concerned, 14 plasma samples from recipients of different ages (47.4 ± 10 years) were tested before and after transplant for Glycotests to evaluate firstly the transplant effects, in comparison with ten not transplanted age‐matched healthy volunteers (age: 47.4 ± 12.3 years), included in our BioBank plasma collection. Then, an enlarged sample of post‐transplanted 36 recipients (age: 50.7 ± 10.2 years) was tested for Glycotests and stratified by age donors (Δ age average: from ‐42 years up to +52 years; three age‐mismatches groups: older, younger and similar to recipient ages). Liver functional markers, that is, ALP, GGT, albumin, ALT, AST, TBIL, and IBIL, were evaluated before and after transplant and some of them (ALT, AST, TBIL, and IBIL) correlated with Glycotests after transplant.

### DNA and RNA extraction

See Data S1.

#### MiRs profiling

It was performed using Applied Biosystems 7900 HT real‐time PCR instrument (Applied Biosystems Inc., Foster City CA, USA) and human miR card A (Applied Biosystems by Life Technologies, NY, USA), containing the most common 365 human miR assays, including those related to liver, in addition to selected small nucleolar RNAs. RNA was converted to cDNA by priming with a mixture of looped primers (MegaPlex kit, Applied Biosystems by Life Technologies) according to manufacturer's instructions. Pre‐amplification was performed using 3 μL of input RNA with PreAmp kit (Life Technologies). 9 μL of pre‐amplified cDNA was used for RT–PCR with the card.

#### RT–qPCR

See Data S1.

#### Telomere length

See Data S1.

#### Gene Expression profiling

See Data S1.

#### *In silico* mirR target prediction

See Data S1.

#### RT–qPCR of mRNA

GLT1, ELL2, and ARDD3 gene transcripts were detected with specific primers See Data S1.

#### Histology and Immunohistochemistry (IHC)

See Data S1.

#### Luciferase and functional assays

See Data S1.

#### Glycotests by DSA‐FACE

See Data S1. The different Glycotests depending on different N‐glycan peaks (see the profiles in Fig. S6) are the following:

- *GlycoAge test*: log ratio of two diantennary, core‐a‐1,6‐fucosylated N‐glycans: NGA2F (or peak 1**)**, agalactosylated, and NA2F (or peak 6), digalactosylated.
- *GlycoCirrho test:* log ratio of NA2FB (or peak 7), a digalacto, core‐a‐1,6‐fucosylated bisecting diantennary glycan, and NA3 (or peak 8), a tri‐galactosylated triantennary glycan.
- *GlycoFibro Test:* log ratio between NGA2FB (or peak 2), an agalacto, core‐a‐1,6‐fucosylated, bisecting diantennary glycan, and NA3 (or peak 8), a tri‐galactosylated triantennary glycan.
- *GlycoNash Test:* log ratio of NGA2F (or peak 1) and NA2 (peak 5) a digalactosylated diantennary glycan.

### Statistical analysis

See Data S1.

## Funding

This work was funded by PRIN2008 to GLG; FP7 EU‐259679‐IDEAL: ‘Integrated research on DEvelopmental determinants of Aging and Longevity’ to CF; FP7 EU‐602757‐HUMAN: ‘Health and the understanding of Metabolism, Aging and Nutrition’ to CF. Further funds from the ‘Università Politecnica delle Marche’ to ADP and FO and in part by Grande Oriente d'Italia (GOI), Massoneria Italiana, Collegio delle Marche.

## Conflicts of interest

All authors declare no conflicts of interest.

## Author Contributions

See Data S1.

## Supporting information


**Fig. S1** The most highly expressed miRs in human liver.

**Fig. S2** Histology Haematoxylin‐Eosin, 10× magnification.

**Fig. S3** (A) Conserved seed sequences of miR‐200c and miR‐31 on GLT1 (SLC1A2) gene. Schematic representation of miR‐200c and miR‐31 binding sites are indicated. The seed sequences are also indicated. The nucleotide (nt) counts start from the beginning of 3′UTR of GLT1 mRNA (NM¬004171.3). (B) Poorly conserved seed sequences of miR‐200c and miR‐31 on GLT1 (SLC1A2) gene. Schematic representation of miR‐200c and miR‐31 binding sites are indicated. The seed sequences are indicated. The nucleotide (nt) counts start from the beginning of 3′UTR of GLT1 mRNA (NM¬004171.3).

**Fig. S4** Poorly conserved seed sequences are not inhibited by miRs over‐expression.

**Fig. S5** Expression of miRs‐31‐5p and 200c‐3p in HepG2 cells after transfection.

**Fig. S6** Typical desialylated N‐glycan profiles from total plasma proteins.

**Fig. S7** Correlation map between standard blood markers of liver function and Glycotests.

**Table S1** (A‐B) Spearman correlation analyses between miRs expression and telomere length and *P* values

**Table S2** (A‐B) The top 76 mRNA most downregulated probes are listed according to the *P* values of log Old/Young (see also Probe ID, name of gene; description of the gene and KEGG pathways)

**Table S3** Standard blood markers of liver function in recipients before and after transplant.

**Data S1** Supplemental materials and methods.
