# Supplementary figures and images for: Identification of miR‐31‐5p, miR‐141‐3p, miR‐200c‐3p, and GLT1 as human liver aging markers sensitive to donor–recipient age‐mismatch in transplants
Source: Aging Cell. 2016 Dec 20;16(2):262–72. doi: 10.1111/acel.12549 (PMC5334540; doi:10.1111/acel.12549)

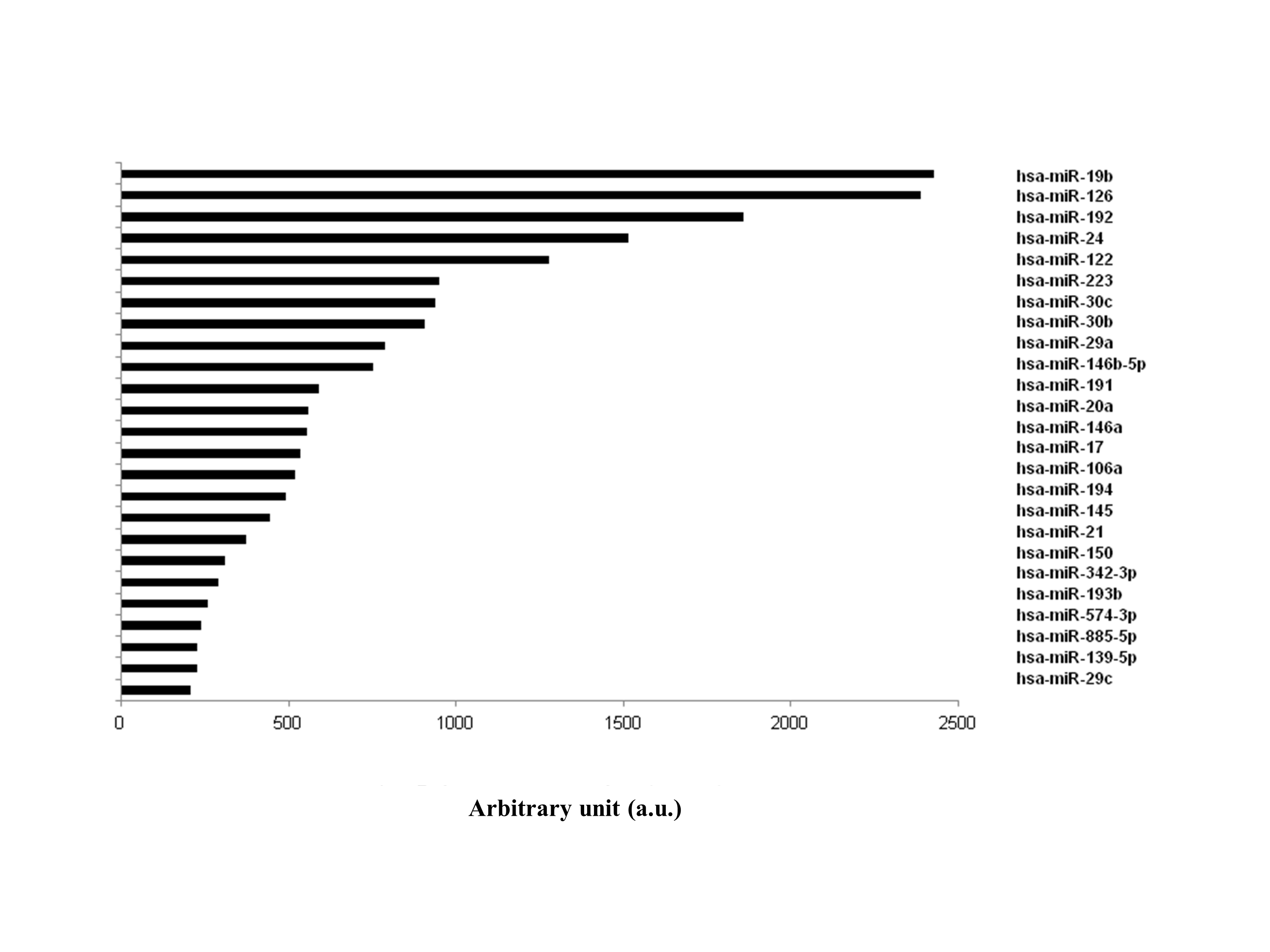

Supplement: Supplementary file 1 — Fig. S1 The most highly expressed miRs in human liver. [file ACEL-16-262-s001.tif]

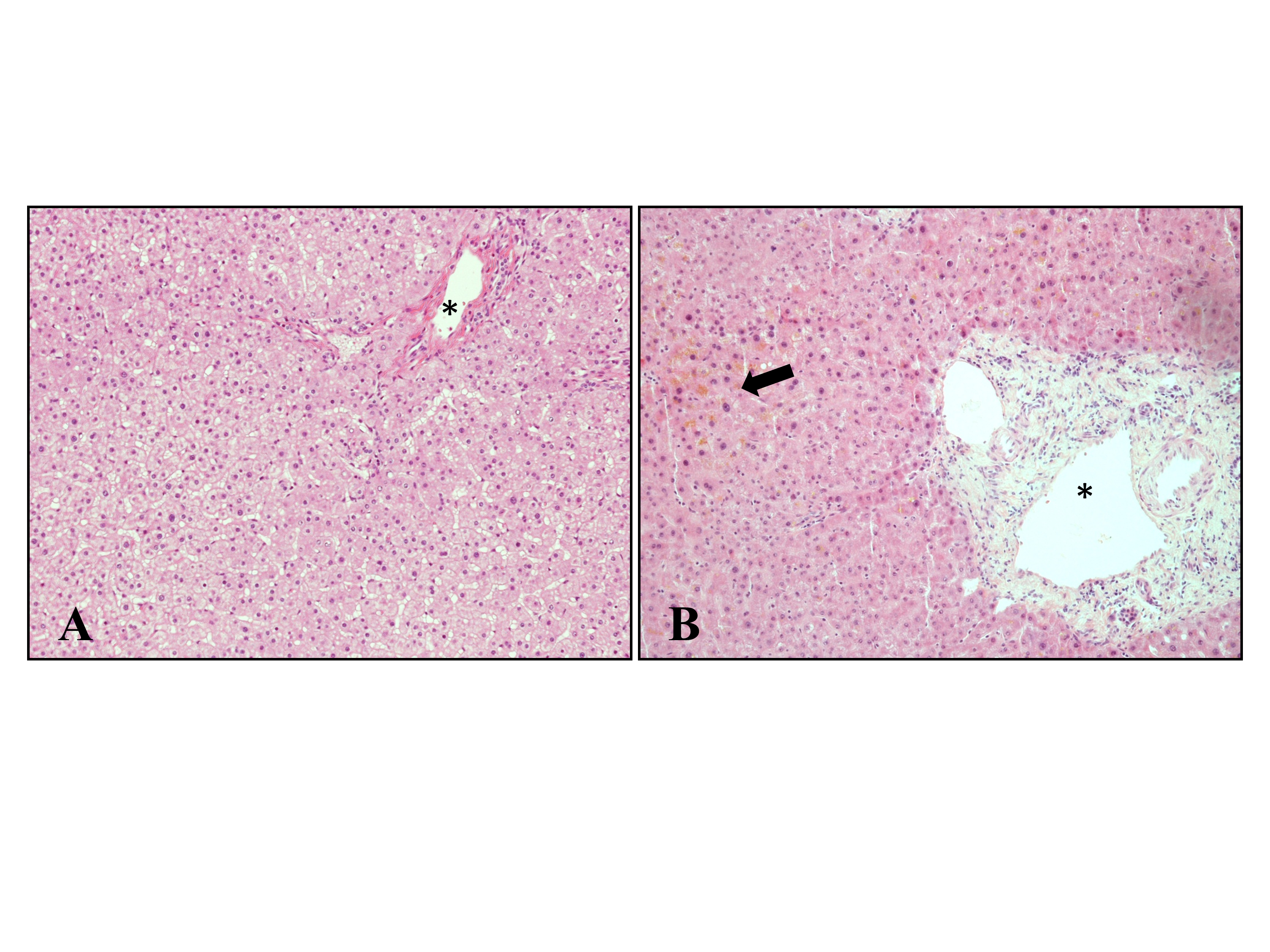

Supplement: Supplementary file 2 — Fig. S2 Histology Haematoxylin‐Eosin, 10× magnification. [file ACEL-16-262-s002.tif]

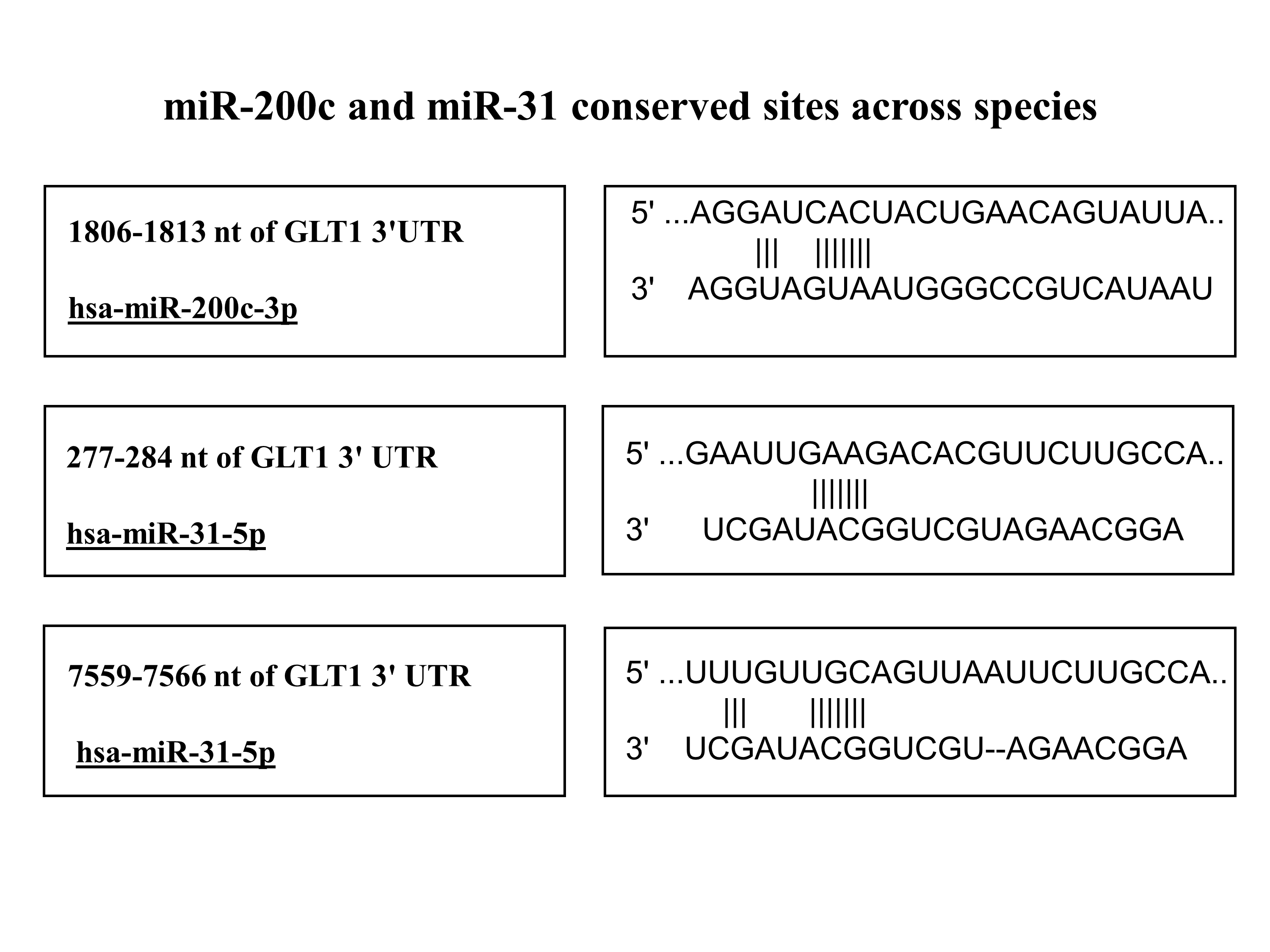

Supplement: Supplementary file 3 — Fig. S3 (A) Conserved seed sequences of miR‐200c and miR‐31 on GLT1 (SLC1A2) gene. Schematic representation of miR‐200c and miR‐31 binding sites are indicated. The seed sequences are also indicated. The nucleotide (nt) counts start from the beginning of 3′UTR of GLT1 mRNA (NM¬004171.3). (B) Poorly conserved seed sequences of miR‐200c and miR‐31 on GLT1 (SLC1A2) gene. Schematic representation of miR‐200c and miR‐31 binding sites are indicated. The seed sequences are indicated. The nucleotide (nt) counts start from the beginning of 3′UTR of GLT1 mRNA (NM¬004171.3). [file ACEL-16-262-s003.tif]

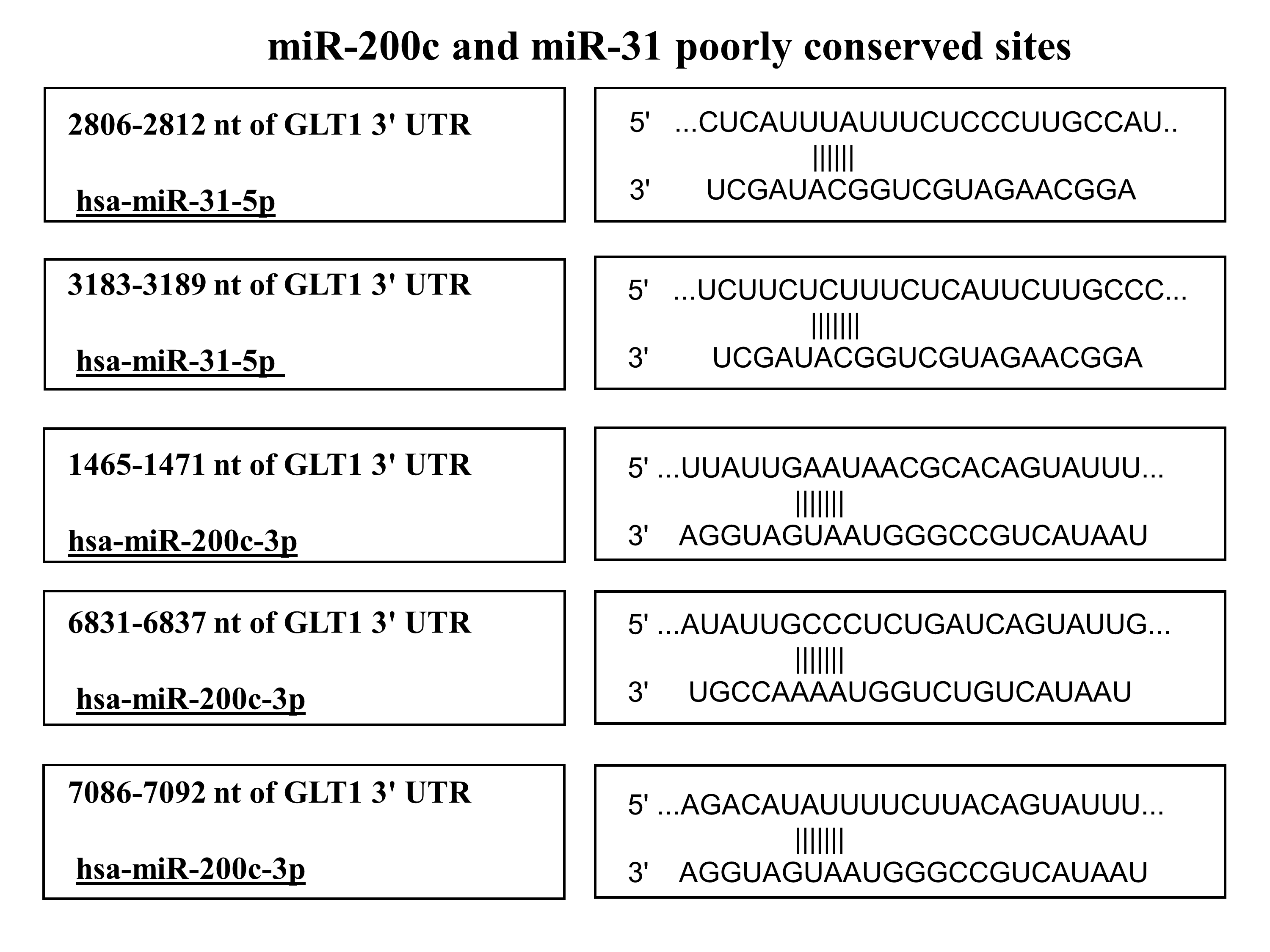

Supplement: Supplementary file 4 [file ACEL-16-262-s004.tif]

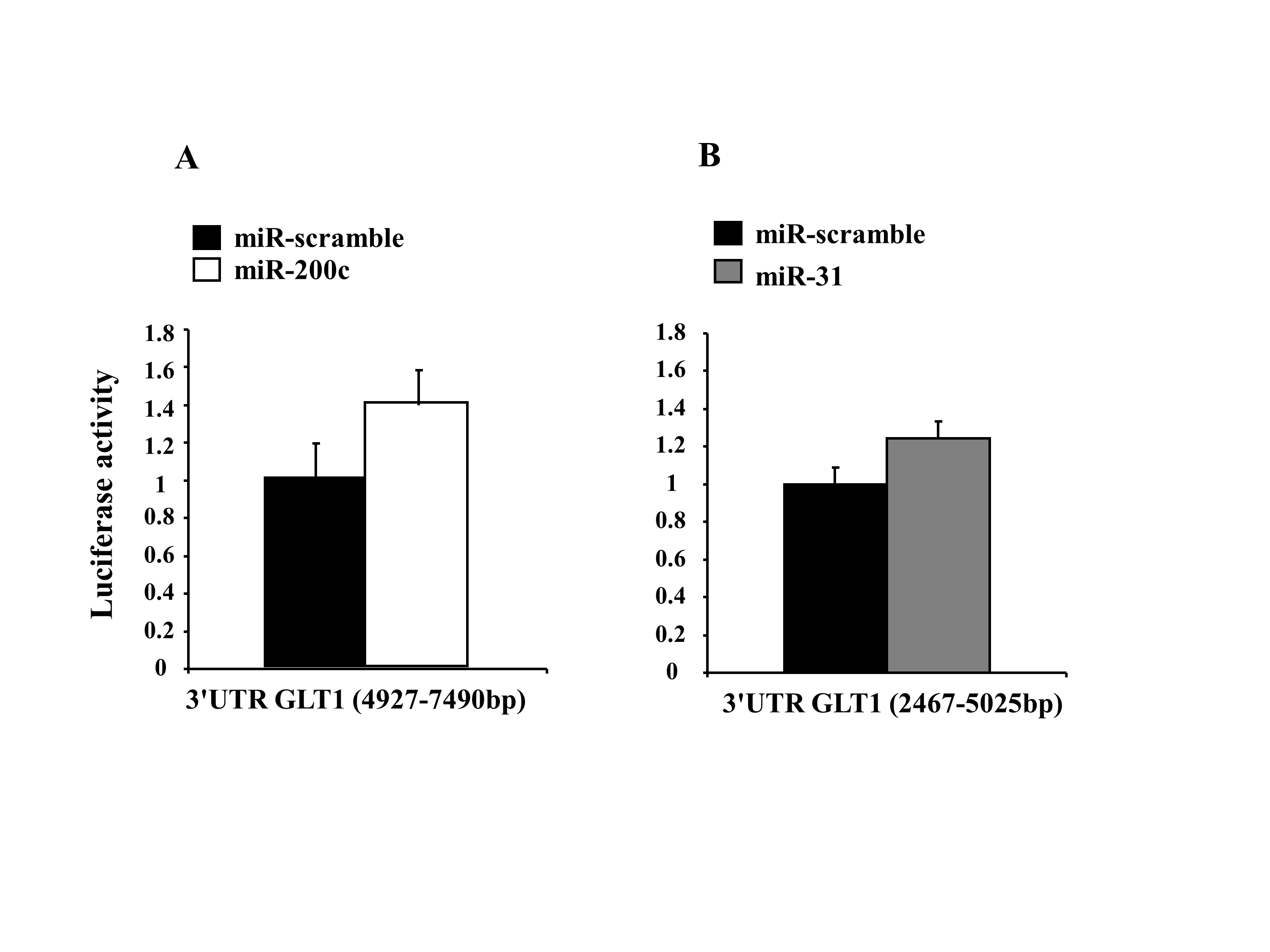

Supplement: Supplementary file 5 — Fig. S4 Poorly conserved seed sequences are not inhibited by miRs over‐expression. [file ACEL-16-262-s005.tif]

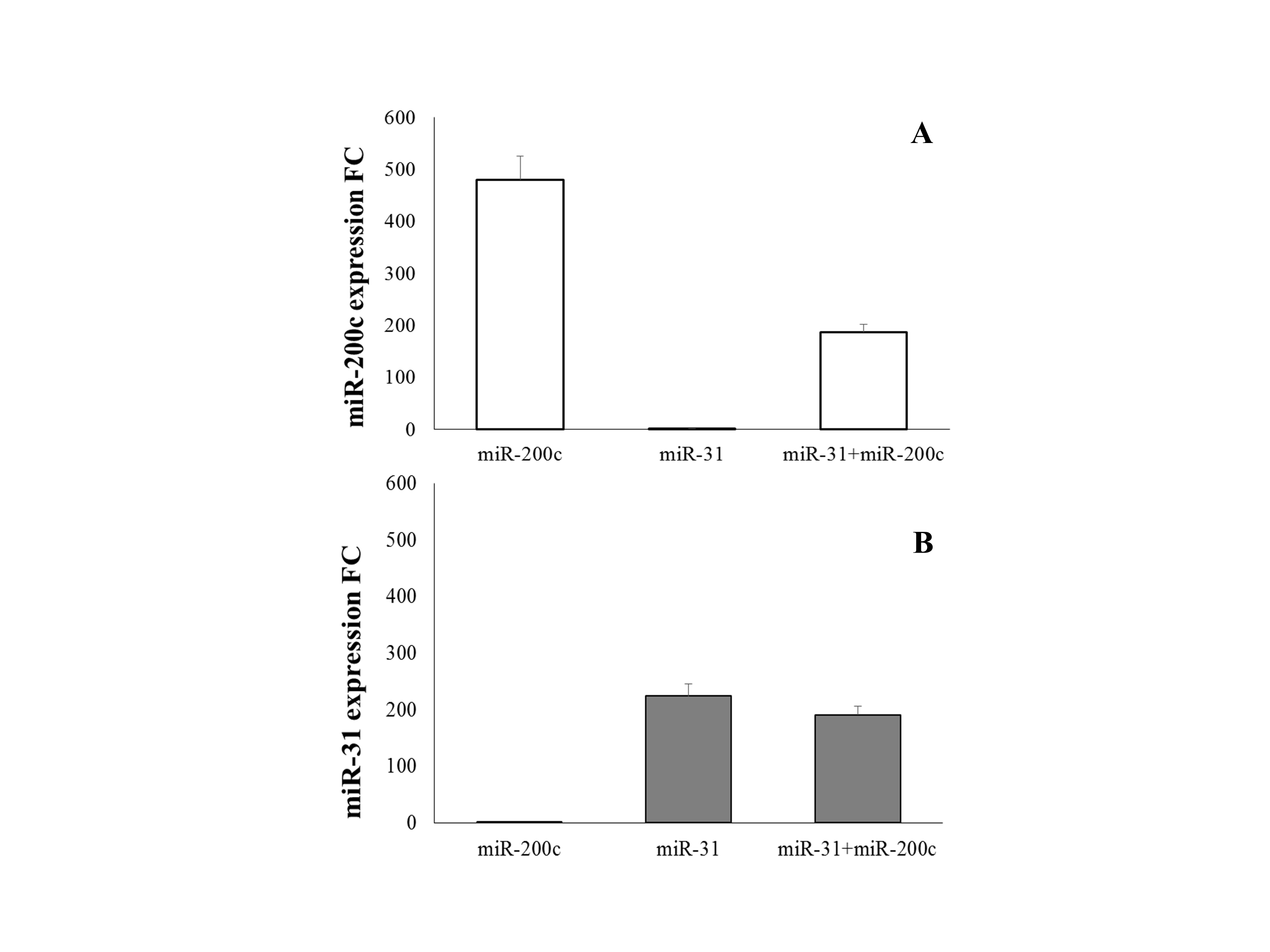

Supplement: Supplementary file 6 — Fig. S5 Expression of miRs‐31‐5p and 200c‐3p in HepG2 cells after transfection. [file ACEL-16-262-s006.tif]

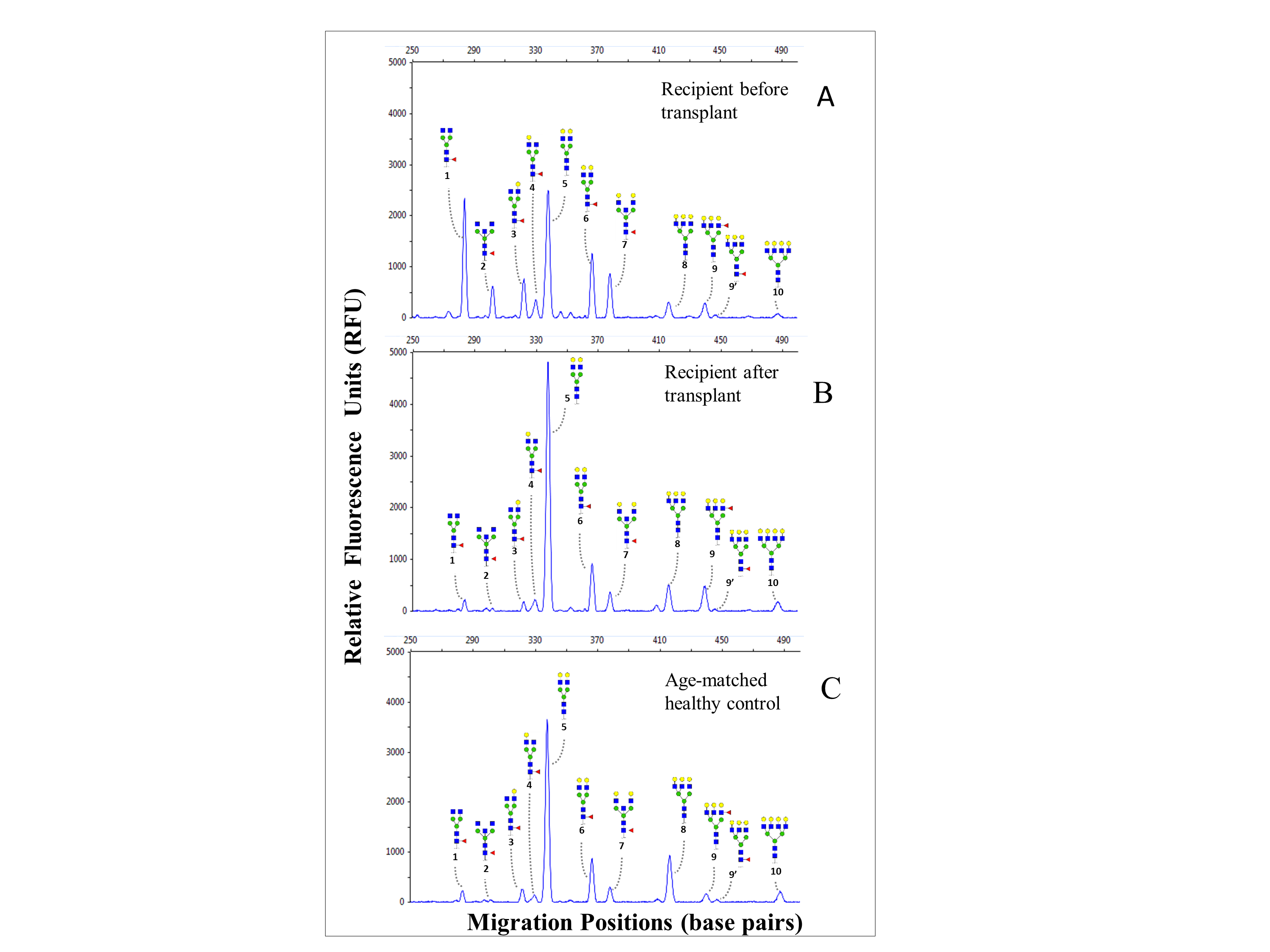

Supplement: Supplementary file 7 — Fig. S6 Typical desialylated N‐glycan profiles from total plasma proteins. [file ACEL-16-262-s007.tif]

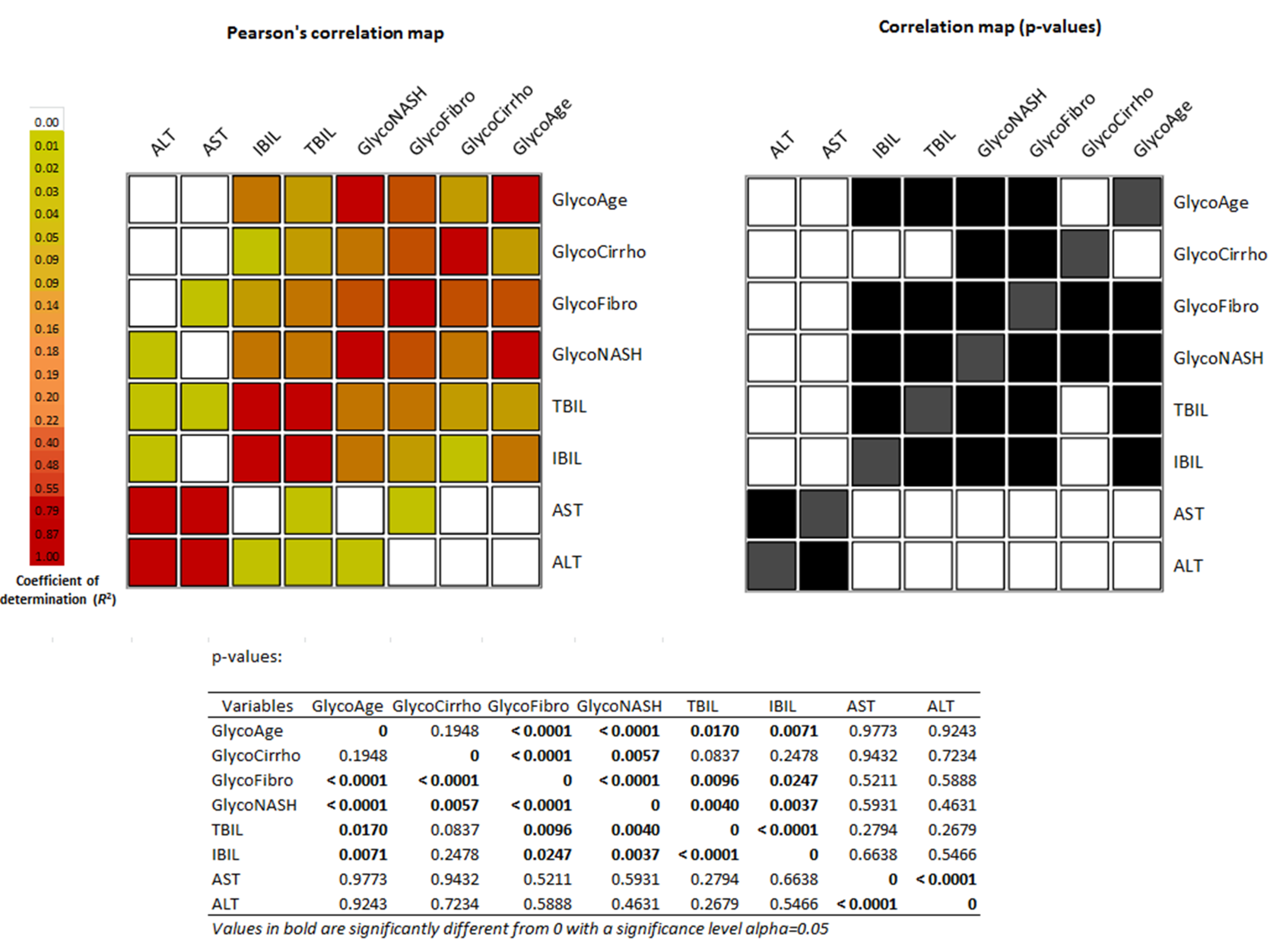

Supplement: Supplementary file 8 — Fig. S7 Correlation map between standard blood markers of liver function and Glycotests. [file ACEL-16-262-s008.tif]

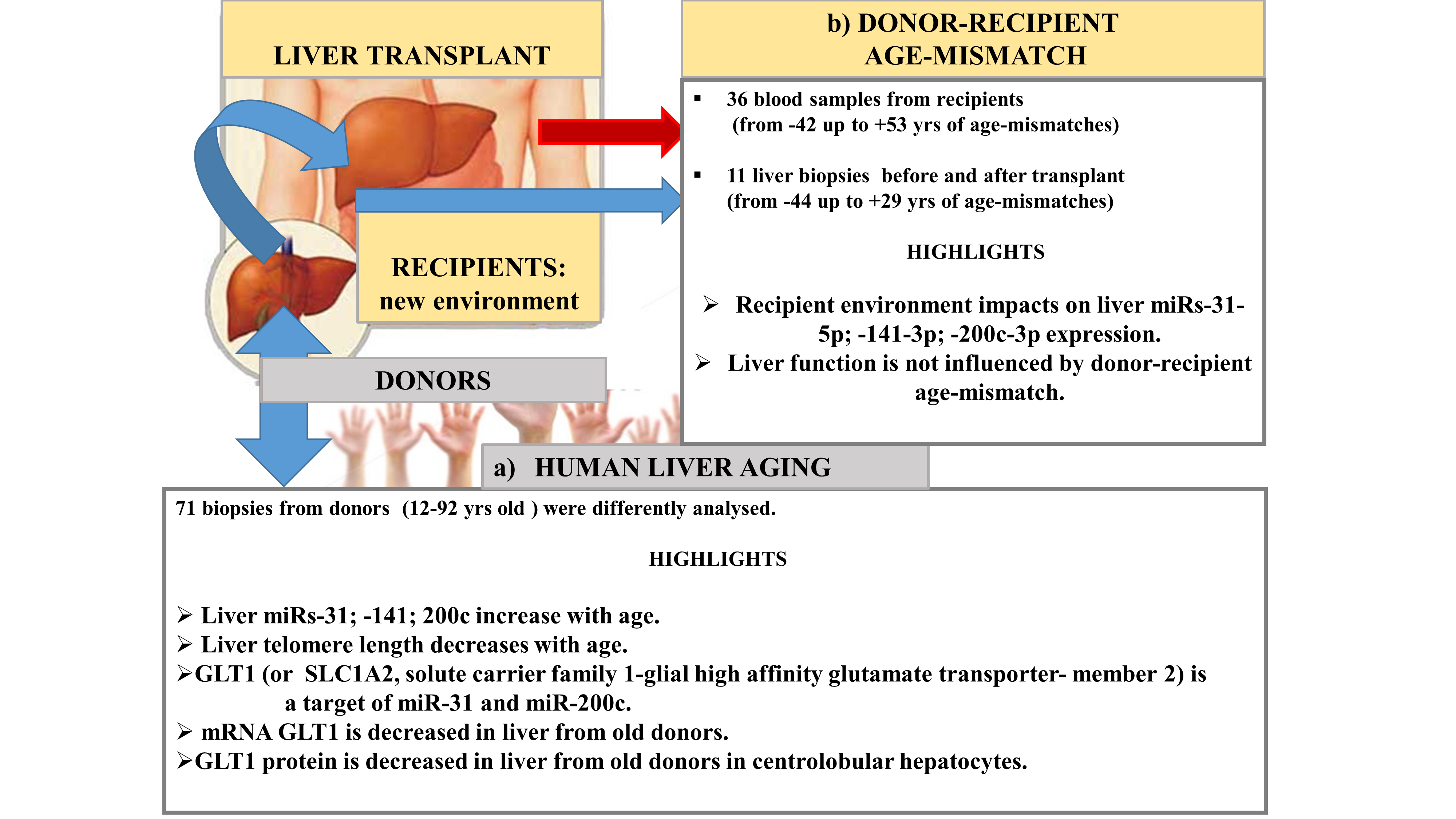

Supplement: Supplementary file 13 [file ACEL-16-262-s013.tif]
