# Supplementary material for: Identification of miR‐31‐5p, miR‐141‐3p, miR‐200c‐3p, and GLT1 as human liver aging markers sensitive to donor–recipient age‐mismatch in transplants
Source: Aging Cell. 2016 Dec 20;16(2):262–72. doi: 10.1111/acel.12549 (PMC5334540; doi:10.1111/acel.12549)
Supplement: Supplementary file 9 — Table S1 (A‐B) Spearman correlation analyses between miRs expression and telomere length and P values [file ACEL-16-262-s009.doc]

**Table 1S-a Spearman correlation coefficients with Age and p values**

**Table 1S-b Spearman correlation coefficients among variables and p values**
