# Supplementary material for: Identification of miR‐31‐5p, miR‐141‐3p, miR‐200c‐3p, and GLT1 as human liver aging markers sensitive to donor–recipient age‐mismatch in transplants
Source: Aging Cell. 2016 Dec 20;16(2):262–72. doi: 10.1111/acel.12549 (PMC5334540; doi:10.1111/acel.12549)
Supplement: Supplementary file 10 — Table S2 (A‐B) The top 76 mRNA most downregulated probes are listed according to the P values of log Old/Young (see also Probe ID, name of gene; description of the gene and KEGG pathways) [file ACEL-16-262-s010.doc]

**Table2S_a: List of the 76 transcripts most down-regulated with age. Probe ID, name of gene (its description), p values, logarithm between old and young groups are reported**

| **ProbeId** | **Gene** | **P value** | **log(O/Y)** | **Description** |
| --- | --- | --- | --- | --- |
| 225491_at | SLC1A2 | 0.00150175 | -1.79726 | solute carrier family 1 (glial high affinity glutamate transporter), member 2 |
| 36711_at | MAFF | 0.0198226 | -1.35002 | v-maf musculoaponeurotic fibrosarcoma oncogene homolog F (avian) |
| 208389_s_at | SLC1A2 | 0.00215235 | -1.11444 | solute carrier family 1 (glial high affinity glutamate transporter), member 2 |
| 235592_at | ELL2 | 0.00797789 | -1.08558 | elongation factor, RNA polymerase II, 2 |
| 206239_s_at | SPINK1 | 0.0386435 | -1.02997 | serine peptidase inhibitor, Kazal type 1 |
| 213332_at | PAPPA2 | 0.0090129 | -1.00324 | pappalysin 2 |
| 228492_at | USP9Y | 0.0342309 | -0.98779 | ubiquitin specific peptidase 9, Y-linked (fat facets-like, Drosophila) |
| 239858_at | TBL1Y | 0.0285865 | -0.949493 | transducin (beta)-like 1Y-linked |
| 217564_s_at | CPS1 | 0.0486184 | -0.930975 | carbamoyl-phosphate synthetase 1, mitochondrial |
| 202627_s_at | SERPINE1 | 0.0285701 | -0.930549 | serpin peptidase inhibitor, clade E, member 1 |
| 228912_at | VIL1 | 0.0286942 | -0.917505 | villin 1 |
| 213122_at | TSPYL5 | 0.02843 | -0.882584 | TSPY-like 5 |
| 242662_at | PCSK6 | 0.0371008 | -0.879523 | proprotein convertase subtilisin/kexin type 6 |
| 229523_at | TTMA | 0.011846 | -0.792103 | two transmembrane domain family member A |
| 219753_at | STAG3 | 0.0320653 | -0.774492 | stromal antigen 3 |
| 219718_at | FLJ10986 | 0.0167371 | -0.747657 | hypothetical protein FLJ10986 |
| 205244_s_at | SLC13A3 | 0.0267584 | -0.742727 | solute carrier family 13 (sodium-dependent dicarboxylate transporter), member 3 |
| 226909_at | KIAA1729 | 0.000266527 | -0.72305 | KIAA1729 protein |
| 213349_at | TMCC1 | 0.0216066 | -0.702065 | transmembrane and coiled-coil domain family 1 |
| 232138_at | MBNL2 | 0.028364 | -0.702029 | muscleblind-like 2 (Drosophila) |
| 203571_s_at | C10orf116 | 0.0198343 | -0.6932 | chromosome 10 open reading frame 116 |
| 1554668_a_at | C1orf179 | 0.0254896 | -0.684637 | chromosome 1 open reading frame 179 |
| 1568920_at | SOX5 | 0.0466449 | -0.678051 | SRY (sex determining region Y)-box 5 |
| 207820_at | ADH1A | 0.0443009 | -0.654654 | alcohol dehydrogenase 1A (class I), alpha polypeptide |
| 207543_s_at | P4HA1 | 0.0175138 | -0.646296 | procollagen-proline, 2-oxoglutarate 4-dioxygenase, alpha polypeptide I |
| 201626_at | INSIG1 | 0.0424591 | -0.643816 | insulin induced gene 1 |
| 221646_s_at | ZDHHC11 | 0.00716987 | -0.600779 | zinc finger, DHHC-type containing 11 |
| 212812_at | SERINC5 | 0.0340322 | -0.592868 | serine incorporator 5 |
| 230372_at |  | 0.0221712 | -0.588044 |  |
| 229596_at | AMDHD1 | 0.00909996 | -0.587223 | amidohydrolase domain containing 1 |
| 222033_s_at | FLT1 | 0.0396139 | -0.584708 | fms-related tyrosine kinase 1 |
| 224831_at | CPEB4 | 0.0460282 | -0.582735 | cytoplasmic polyadenylation element binding protein 4 |
| 220114_s_at | STAB2 | 0.0354378 | -0.582411 | stabilin 2 |
| 1554945_x_at | VIL1 | 0.0280724 | -0.577537 | villin 1 |
| 225664_at | COL12A1 | 0.0314264 | -0.574768 | collagen, type XII, alpha 1 |
| 238160_at | ACOT12 | 0.0172918 | -0.570613 | acyl-CoA thioesterase 12 |
| 202497_x_at | SLC2A3 | 0.0149783 | -0.566671 | solute carrier family 2 (facilitated glucose transporter), member 3 |
| 210058_at | MAPK13 | 0.0356534 | -0.562216 | mitogen-activated protein kinase 13 |
| 203716_s_at | DPP4 | 0.000571254 | -0.560789 | dipeptidyl-peptidase 4 (CD26, adenosine deaminase complexing protein 2) |
| 223427_s_at | EPB41L4B | 0.0348888 | -0.554063 | erythrocyte membrane protein band 4.1 like 4B |
| 239156_at | BCAR3 | 0.0314371 | -0.544763 | breast cancer anti-estrogen resistance 3 |
| 206170_at | ADRB2 | 0.0171514 | -0.536152 | adrenergic, beta-2-, receptor, surface |
| 224367_at | BEX2 | 0.0393039 | -0.535553 | brain expressed X-linked 2 |
| 232472_at | FNDC3B | 0.0143025 | -0.531482 | fibronectin type III domain containing 3B |
| 209109_s_at | TSPAN6 | 0.0495054 | -0.529264 | tetraspanin 6 |
| 205193_at | MAFF | 0.0224397 | -0.522578 | v-maf musculoaponeurotic fibrosarcoma oncogene homolog F (avian) |
| 204159_at | CDKN2C | 0.0275508 | -0.521553 | cyclin-dependent kinase inhibitor 2C (p18, inhibits CDK4) |
| 205478_at | PPP1R1A | 0.0123995 | -0.507809 | protein phosphatase 1, regulatory (inhibitor) subunit 1A |
| 231828_at | LOC253039 | 0.0250936 | -0.497765 | hypothetical protein LOC253039 |
| 218280_x_at | HIST2H2AA3 | 0.0428017 | -0.493925 | histone cluster 2, H2aa3 |
| 227112_at | TMCC1 | 0.0126776 | -0.49315 | transmembrane and coiled-coil domain family 1 |
| 225745_at | LRP6 | 0.0364667 | -0.492527 | low density lipoprotein receptor-related protein 6 |
| 1559975_at | BTG1 | 0.00959891 | -0.491492 | B-cell translocation gene 1, anti-proliferative |
| 228717_at | PANK1 | 0.0468439 | -0.482847 | pantothenate kinase 1 |
| 216025_x_at | CYP2C9 | 0.0306399 | -0.474365 | cytochrome P450, family 2, subfamily C, polypeptide 9 |
| 230971_x_at | LOC388323 | 0.022533 | -0.46775 | hypothetical LOC388323 |
| 220017_x_at | CYP2C9 | 0.0131229 | -0.464421 | cytochrome P450, family 2, subfamily C, polypeptide 9 |
| 207262_at | APOF | 0.0146445 | -0.462648 | apolipoprotein F |
| 212646_at | RFTN1 | 0.00570733 | -0.460539 | raftlin, lipid raft linker 1 |
| 214846_s_at | ALPK3 | 0.014797 | -0.453455 | alpha-kinase 3 |
| 204432_at | SOX12 | 0.0433211 | -0.45298 | SRY (sex determining region Y)-box 12 |
| 227598_at | LRRC61 | 0.0314587 | -0.447592 | leucine rich repeat containing 61 |
| 229764_at | FAM79B | 0.0379709 | -0.443482 | family with sequence similarity 79, member B |
| 200921_s_at | BTG1 | 0.0335465 | -0.441596 | B-cell translocation gene 1, anti-proliferative |
| 230848_s_at | MGA | 0.0264586 | -0.435596 | MAX gene associated |
| 238448_at | MRPL19 | 0.0376885 | -0.434457 | mitochondrial ribosomal protein L19 |
| 235129_at | PPP1R1A | 0.0300147 | -0.430529 | protein phosphatase 1, regulatory (inhibitor) subunit 1A |
| 223681_s_at | INADL | 0.00973513 | -0.429733 | InaD-like (Drosophila) |
| 203560_at | GGH | 0.0426514 | -0.42824 | gamma-glutamyl hydrolase (conjugase, folylpolygammaglutamyl hydrolase) |
| 203711_s_at | HIBCH | 0.0184021 | -0.424071 | 3-hydroxyisobutyryl-Coenzyme A hydrolase |
| 242963_at | MGC26963 | 0.0423747 | -0.423453 | sphingomyelin synthase 2 |
| 224797_at | ARRDC3 | 0.0374855 | -0.415766 | arrestin domain containing 3 |
| 206643_at | HAL | 0.00713798 | -0.410941 | histidine ammonia-lyase |
| 209967_s_at | CREM | 0.0459381 | -0.408696 | cAMP responsive element modulator |
| 218724_s_at | TGIF2 | 0.0412593 | -0.405523 | TGFB-induced factor homeobox 2 |
| 209420_s_at | SMPD1 | 0.0190583 | -0.404244 | sphingomyelin phosphodiesterase 1, acid lysosomal (acid sphingomyelinase) |

**Table2S_b: probe ID and KEGG pathway annotation**

| **ProbeId** | **Kegg Pathway** |
| --- | --- |
| 225491_at | 05030: Amyotrophic lateral sclerosis (ALS) |
| 36711_at |  |
| 208389_s_at | 05030: Amyotrophic lateral sclerosis (ALS) |
| 235592_at |  |
| 206239_s_at |  |
| 213332_at |  |
| 228492_at |  |
| 239858_at | 04310: Wnt signaling pathway |
| 217564_s_at | 00220: Urea cycle and metabolism of amino groups, 00251: Glutamate metabolism, 00330: Arginine and proline metabolism, 00910: Nitrogen metabolism |
| 202627_s_at | 04610: Complement and coagulation cascades |
| 228912_at |  |
| 213122_at |  |
| 242662_at |  |
| 229523_at |  |
| 219753_at |  |
| 219718_at |  |
| 205244_s_at |  |
| 226909_at |  |
| 213349_at |  |
| 232138_at |  |
| 203571_s_at |  |
| 1554668_a_at | |
| 1568920_at |  |
| 207820_at | 00010: Glycolysis / Gluconeogenesis, 00071: Fatty acid metabolism, 00120: Bile acid biosynthesis, 00350: Tyrosine metabolism, 00561: Glycerolipid metabolism, 00624: 1- and 2-Methylnaphthalene degradation, 00980: Metabolism of xenobiotics by cytochrome P450 |
| 207543_s_at | 00330: Arginine and proline metabolism |
| 201626_at |  |
| 221646_s_at |  |
| 212812_at |  |
| 230372_at |  |
| 229596_at |  |
| 222033_s_at | 04060: Cytokine-cytokine receptor interaction, 04510: Focal adhesion |
| 224831_at |  |
| 220114_s_at |  |
| 1554945_x_at | |
| 225664_at |  |
| 238160_at | 00620: Pyruvate metabolism |
| 202497_x_at |  |
| 210058_at | 04010: MAPK signaling pathway, 04370: VEGF signaling pathway, 04620: Toll-like receptor signaling pathway, 04664: Fc epsilon RI signaling pathway, 04670: Leukocyte transendothelial migration, 04912: GnRH signaling pathway, 05120: Epithelial cell signaling in Helicobacter pylori infection |
| 203716_s_at |  |
| 223427_s_at |  |
| 239156_at |  |
| 206170_at | 04020: Calcium signaling pathway, 04080: Neuroactive ligand-receptor interaction |
| 224367_at |  |
| 232472_at |  |
| 209109_s_at |  |
| 205193_at |  |
| 204159_at | 04110: Cell cycle |
| 205478_at | 04720: Long-term potentiation |
| 231828_at |  |
| 218280_x_at |  |
| 227112_at |  |
| 225745_at | 04310: Wnt signaling pathway |
| 1559975_at |  |
| 228717_at | 00770: Pantothenate and CoA biosynthesis |
| 216025_x_at | 00590: Arachidonic acid metabolism, 00591: Linoleic acid metabolism, 00902: Monoterpenoid biosynthesis, 00903: Limonene and pinene degradation, 00980: Metabolism of xenobiotics by cytochrome P450 |
| 230971_x_at |  |
| 220017_x_at | 00590: Arachidonic acid metabolism, 00591: Linoleic acid metabolism, 00902: Monoterpenoid biosynthesis, 00903: Limonene and pinene degradation, 00980: Metabolism of xenobiotics by cytochrome P450 |
| 207262_at |  |
| 212646_at |  |
| 214846_s_at |  |
| 204432_at |  |
| 227598_at |  |
| 229764_at |  |
| 200921_s_at |  |
| 230848_s_at |  |
| 238448_at |  |
| 235129_at | 04720: Long-term potentiation |
| 223681_s_at | 04530: Tight junction |
| 203560_at | 00790: Folate biosynthesis |
| 203711_s_at |  |
| 242963_at | 00600: Sphingolipid metabolism |
| 224797_at |  |
| 206643_at | 00340: Histidine metabolism, 00910: Nitrogen metabolism |
| 209967_s_at |  |
| 218724_s_at |  |
| 209420_s_at | 00600: Sphingolipid metabolism |
