# Supplementary material for: Identification of miR‐31‐5p, miR‐141‐3p, miR‐200c‐3p, and GLT1 as human liver aging markers sensitive to donor–recipient age‐mismatch in transplants
Source: Aging Cell. 2016 Dec 20;16(2):262–72. doi: 10.1111/acel.12549 (PMC5334540; doi:10.1111/acel.12549)
Supplement: Supplementary file 11 — Table S3 Standard blood markers of liver function in recipients before and after transplant. [file ACEL-16-262-s011.doc]

**Table S3.** Standard liver blood markers detected in recipients at pre and post-transplant time.

|  |  | **Pre**  **Transplant** | **Post**  **Transplant** |  |
| --- | --- | --- | --- | --- |
| Liver Markers | Couples (N) | Mean (SD) | Mean (SD) | Significance (p value)* |
| Albumin (g/100mL) | 20 | 4.00 (0.66) | 4.07 (0.42) | 0.632 |
| TBIL (µmol/L) | 36 | 7.40 (10.46) | 0.86 (0.56) | **0.0005** |
| IBIL (µmol/L) | 36 | 2.27 (2.43) | 0.48 (0.35) | **<0.0001** |
| AST (IU/L) | 36 | 47.06 (49.57) | 108.25 (220.5) | 0.121 |
| ALT (IU/L) | 36 | 115.75 (354.75) | 49.19 (48.77) | 0.2846 |
| GGT (IU/L) | 12 | 51.58 (49.55) | 113.92 (150.10) | 0.097 |
| ALP (IU/L) | 12 | 207.17 (69.53) | 218.83 (110.62) | 0.773 |

* paired samples t-Test
