# Supplementary material for: Identification of miR‐31‐5p, miR‐141‐3p, miR‐200c‐3p, and GLT1 as human liver aging markers sensitive to donor–recipient age‐mismatch in transplants
Source: Aging Cell. 2016 Dec 20;16(2):262–72. doi: 10.1111/acel.12549 (PMC5334540; doi:10.1111/acel.12549)
Supplement: Supplementary file 12 — Data S1 Supplemental materials and methods. [file ACEL-16-262-s012.doc]

**Identification of miRs-31, -141, 200c and GLT1 as human liver aging-markers sensitive to donor-recipient age-mismatch in transplants**

***Supplemental materials and methods***

**DNA and RNA extraction**. High molecular weight DNA was extracted by Qiagen kit QiAmp mini Kit®, (Qiagen, Milan, Italy) following manufacturer’s instructions. About 5 μg of DNA were obtained from 20 mg of tissue. RNA extraction was obtained by mirVana® miRNA Isolation Kit (Ambion Inc, Austin, Texas, USA), which allows isolation of both enriched miR and larger RNA species, following manufacturer’s instructions. Total RNA quantity was quantified using Nanodrop 1000 (Thermo Fisher Scientific, Freemont, CA, USA); purity/integrity by 2100 Bioanalyzer (Agilent Technologies, Palo Alto, CA, USA).

**RT-qPCR***.* Single specific miR expression was measured on 45 liver biopsies with the TaqMan miR reverse transcription kit and TaqMan MicroRNA Assay (Applied Biosystems by Life Technologies, NY, USA) with some modifications. Briefly, total RNA was reverse transcribed (RT) by TaqMan MicroRNA RT kit. 5 μl of RT reactions contained 1 μl of each miR specific stem-loop primers and 1.67 μl of input RNA. The mixture was incubated at 16°C for 30’, 42°C for 30’ and 85°C for 5’. Subsequently real-time qPCR was performed. 20 μl of PCR reaction: 1 μl of 20x Taqman MicroRNA Assay containing PCR primers and probes (5’-FAM), 10 μl of 2x TaqMan Universal Master mix no UNG (Life Technologies) and 5 μl of RT product were mixed. The reaction was first incubated at 95°C for 2’ followed by 40 cycles of 95°C for 15’’ and 60°C for 1’. MiRs were quantified using TaqMan miR assays (Life Technologies), according to the manufacturer’s protocol. Data were analysed with the RT-qPCR OpticonMonitor version 2 (MJ Research) with automatic setting for assigning baseline. The Ct values of qPCR assays greater than 35 were considered not expressed. MiR fold changes were calculated by delta Ct method and normalised to RNU-44. All assays were performed in duplicate.

**In silico mirR target prediction**. TargetScan database (http://www.targetscan.org/) was used to identify the predicted target genes of the miRs. For each miR a dataset of the predicted targeted genes was created, i.e. a group list of the target Refseq IDs. Refseq IDs have been automatically indexed using a simple Fortran written program (SID1.0; String IDentifier) that looks for IDs shared by the different datasets (Albertini et al., 2011). Furthermore, this approach allowed us to couple with transcriptomic data to identify putative miR targets among the most down-regulated mRNAs in liver from old donors.

**Telomere length**. It was measured as abundance of telomeric template (T) vs a single gene copy (S; 36B4) by qPCR, as previously described (Cawthon et al., 2002). Measurements were performed in triplicate and reported as T/S ratio with respect to a standard sample (human genomic DNA from Roche). All PCR were performed on real-time iCicler (Bio-Rad, Hercules, CA, Italy). Telomere length was also assessed in 45 donors and 11 recipients after transplant.

**Gene Expression profiling**. cDNA synthesis, biotin-labelled target synthesis, 12 HG-U133 plus 2.0 GeneChip (Affymetrix, Santa Clara, CA, USA) arrays hybridization (for 12 biopsies), staining and scanning were performed according to the standard protocol supplied by Affymetrix. Probe cell intensity data (CEL file generation) as well as array registration, data management, instrument and workflow management were conducted using Affymetrix® GeneChip® Command Console® Software (AGCC). The probe level data were converted to expression values using the Robust Multiarray Average (RMA) procedure. R-AffyQC Report, R-Affy-PLM, R-RNA Degradation Plot and QC tools, integrated in Partek GS®, were used to perform all data quality controls. Generated data were MIAMI-compliant. One chip was excluded after principal component analysis (PCA).

**RT-qPCR of mRNA**. GLT1, ELL2 and ARDD3 gene transcripts were assessed with the following primers:

1. GLT1 (or SLC1A2):

Sense 5'AGCAGGCTGATGTCCTCGT3'
Antisense  5'GATTCCGTCCTTCCTGTTGGA3'

1. ELL2:

Sense 5'TGACTGCATCCAGCAAACAT3'

Antisense 5'TCGTTTGTTGCACACACTGTAA3'

1. ARDD3 primers:

Sense 5'ATAGACCATCCTGCCCCTCT3'

Antisense 5'TCAAGCATGTTGGAGCAGTC3'

Data were normalized to GAPDH expression level and reported as mean ± SD. The relative amount of each mRNA was calculated using the comparative threshold (Ct) method with ΔCt = Ct (mRNA)−Ct (GAPDH) and relative quantification of mRNA expression was calculated with the 2^−ΔΔCt^ method. Primers were designed to span introns where possible, using Primer 3 online program (http://frodo.wi.mit.edu/cgi-bin/ primer3primer3_www.cgi).

**Histology and Immunohistochemistry (IHC)** Histological evaluation on liver frozen sections was performed according to the previously reported protocol (Fiorentino et al., 2009, Vasuri et al., 2010, Bellavista et al., 2014) applied at S. Orsola-Malpighi Hospital.

Biopsies were also formalin fixed, paraffin embedded and routinely processed. Two-μm-thick sections were cut and stained with Haematoxylin-Eosin. Graft suitability for transplantation was confirmed in all cases, and the following histological parameters were collected: stage of fibrosis, hepatocyte polymorphism, and lipofuscin accumulation. Formalin-fixed paraffin embedded (FFPE) 2-µm-thick sections were rehydrated through graded steps of ethanol absolute (Xylol 30min, 100% 10 min, 95% 5 min, 70% 5 min). Immunostaining of GLT1 was performed according to the manufacturer's instructions. After dewaxing, antigen retrieval was carried out with microwave method at 100°C for 4 cycles 5 min, in a citrate buffer solution (pH 6.0) and cooling for 20 min. Endogenous peroxidase activity was blocked with 3% H_2_O_2_ in absolute methanol for 10 min at room temperature (rt), in the dark. Sections were incubated in a wet chamber at 4°C with the antibody anti-GLT1 (anti-rabbit polyclonal ab, Novus Biologicals, USA; dilution 1:50) for 1 h, then with EnVision® polymer (DAKO, Glostrup, Denmark) for 20 min at rt. Cell nuclei were stained with Mayer’s haematoxylin (Sigma Chemicals). Negative controls were obtained omitting the primary antibody. Due to the focal expression observed in all liver samples, which proved to be variably weak or strong in periportal/centrilobular zones (lobule zones 1 and 2) and almost always strong in perivenular zone (lobule zone 3), GLT1 expression was assessed by counting and evaluating each periportal and perivenular area in all samples and separately scoring their staining intensity.

**Luciferase and functional assays**. HEK 293 cells were plated in 12-well plates and were transfected with 0,5 μg of the full-length 3’-UTR luciferase constructs or empty vector (pEZX-MT06, pEZX-MT06-(1-2564bp) GLT1 (SLC1A2) 3’UTR, pEZX-MT06-(2467-5025) GLT1 3’UTR, pEZX-MT06-(4927-7490) GLT1 3’UTR, pEZX-MT06-(7385.9689) GLT1 3’UTR) together with 0.25 μg of plKO.1-pre-miR-200c or plKO.1-scramble or 0,25 μg pCMV-MIR/GFP-pre-miR-31 or pCMV-MIR/GFP. Cellular extracts were tested with Dual Luciferase Assay (Promega, Madison, WI, USA) according to the manufacturer instructions, using a Victor 3 1420 Multilabel Counter (Perkin Elmer, Waltham, MA, USA). Values were normalized according to renilla luciferase activity.

HepG2, human hepatocellular carcinoma cell (obtained from ATCC, USA) line was used in this study. HepG2 cells were grown in DMEM medium supplemented with 2 mM L-glutamine, penicillin (20 units/mL), streptomycin (20 μg/mL), and 10% (vol/vol) heat-inactivated fetal calf serum at 37 °C in a saturated humidity atmosphere containing 5% CO2. Transient transfection of miRNA-200c, -31 and empty vector control (Origene Technology, Inc., Rockville, MD), was performed using TransIT®-LT1 Transfection Reagent (Mirus Bio LLC; Madison, WI), according to the manufacturer’s instructions. In brief, 8 × 10^4^ cells were plated in six-well plates and kept overnight for attachment. The next day, cells were transfected with miR-200c mimic, miR-31 mimic or empty vector control. The transfection method was optimised testing different quantities of reagent and miR. In particular, TransIT®-LT1 Transfection Reagent (microlitre)/miR (microgram) ratios of 3:1 were found as optimal. It is important to underline that TransIT®-LT1 Transfection Reagent –miR complex was prepared in serum-free medium. Analyses were performed 72 h after transfection.

**Glycotests by DSA-FACE**. N-glycans were enzymatically released from 5 uL of plasma by using PNGase F (New England Biolabs) before a denaturation step of total plasma proteins as previously described (Vanhooren et al., 2007, cited in the main text). The released plasma N-glycans were then derivatized with the fluorophore 8-amino-1,3,6-pyrenetrisulfonic acid (APTS, Molecular Probes, Eugene, OR) by using a specific buffer (1:1 mixture (v/v) of 20 mM APTS in 1.2 M citric acid and 1 M NaCNBH3 in DMSO), then an incubation at 37°C for 16 h was performed. The reaction was stopped by adding 200 µL of water to each well. Labeled N-glycans were desialylated by using neuraminidase (Arthrobacter ureafaciens, Roche, Germany). 10 μL of APTS labeled and desialylated glycans were analyzed using a capillary electrophoresis (CE)-based ABI 3730xl DNA sequencer (Applied Biosystem) equipped with a standard 36-cm capillary array filled with the POP-7 polyacrylamide linear polymer. Data were processed with PEAK SCANNER software (Applied Biosystem). For each sample, the intensities (heights) of the peaks of the electropherogram were normalized to the sum of the heights of all peaks. The peaks are 10, everyone corresponding to a specific N-glycan structure (see a representative analysis in figure S6) which are used to obtain different Glycotests,

**Statistical analysis**. MiRs profiling was normalised using the median of the overall miR expression on each array (ΔCt). MiRs showing a difference in ΔCt mean between age groups higher than 1.5 (ΔΔCt > 1.5 and < -1.5) were selected. Fold-change (2^-ΔΔCT^) was calculated based on the estimated mean difference. MiR and mRNA expression and telomere length between groups (old *vs* young, donor *vs* recipient) were tested for significance (p < 0.05) by independent t Student test. Correlation analysis of miR and/or telomere length as function of age was determined by Spearman's correlation coefficient. mRNA expression datasets were firstly analysed by PCA and one sample was excluded. The liver transcriptome of 5 young male donors (age ≤ 30 yrs) was compared to that of 6 male old donors (age ≥ 70 yrs). Student's t test was performed for each probe (“mattest” function, Matlab software), and the log ratio was calculated as the average (logarithm) expression of old group minus the average (logarithm) expression of young group. A p value <0.05 was considered significant, and probes were ranked by their log ratio. The top 76 most down-regulated probes were considered for further analysis. IHC scores were assigned to cell areas in distinct zones of the specimen (periportal/centrilobular and perivenular zones) based on color level by visual inspection. Non parametric Mann-Whitney test was applied and p value<0.05 was considered significant. Glycotests were tested by non parametric Wilcoxon signed-ranks and Kruscal Wallis tests for paired and independent samples, respectively. Glycotest correlation with functional liver markers was performed by Pearson and coefficient of determination (R^2^). P value < 0.05 was considered significant. Excel and SPSS v21 were also used for data analysis and visualisation.

**Contribution of Authors**:

- Study concept and design: G L G, M Ce, M C and C F

- Acquisition of data at four Universities (Bologna, Modena, Ancona and Rome, Italy): R L, L G, M C A, AS, F B, E T, E T, C L, V B, C M, F V, A M, M d’A, M C. C.

- Analysis and interpretation of data: D R, F O and M Ca

- Donors/Recipient recruitment: M Ce, A D, S P, C L, E B, G L G

- Drafting of the manuscript; M C, AD P, F O

- Critical revision of the manuscript: M C, G L G, A D’E-G, E B, G P, C M, M Ce, C F.

- Obtained funding; G L Gr & M C; F O & A D P, D R, C F

**References:**

Bellavista E, Martucci M, Vasuri F, Santoro A, Mishto M, Kloss A, Capizzi E, Degiovanni A, Lanzarini C, Remondini D, Dazzi A, Pellegrini S, Cescon M, Capri M, Salvioli S, D’Errico-Grigioni A, Dahlmann B, Grazi GL, Franceschi C (2014) Lifelong maintenance of composition, function and cellular/subcellular distribution of proteasomes in human liver. *Mech. Aging Dev.* **141–142**, 26–34.

Cawthon RM (2002) Telomere measurement by quantitative PCR. *Nucleic Acid* *Res*. **30**, e47.

Fiorentino M, Vasuri F, Ravaioli M, Ridolfi L, Grigioni WF, Pinna AD, D’Errico-Grigioni A (2009) Predictive value of frozen-section analysis in the histological assessment of steatosis before liver transplantation. *Liver Transpl*. **15**, 1821–1825.

Vasuri F, Capizzi E, Bellavista E, Mishto M, Santoro A, Fiorentino M, Capri M, Cescon M, Grazi GL, Grigioni WF, D’Errico-Grigioni A, Franceschi C (2010) Studies on immunoproteasome in human liver. Part I: absence in fetuses, presence in normal subjects, and increased levels in chronic active hepatitis and cirrhosis. *Biochem. Biophys. Res. Commun.* **397**, 301–306.
