# Supplementary material for: Identification of miR‐31‐5p, miR‐141‐3p, miR‐200c‐3p, and GLT1 as human liver aging markers sensitive to donor–recipient age‐mismatch in transplants
Source: Aging Cell. 2016 Dec 20;16(2):262–72. doi: 10.1111/acel.12549 (PMC5334540; doi:10.1111/acel.12549)
Supplement: Supplementary file 14 [file ACEL-16-262-s014.pdf]

| DONOR LIVERS | GENDER M/F | AGE | Cause of death | miRs Profiling | miRNAs validation | Telomer length | Affy Transcriptome | mRNA | Donor-recipient age-mismatch miRs | Donor-recipient age-mismatch Telomere length | IHC |
|---|---|---|---|---|---|---|---|---|---|---|---|
| 88 | M | **12** | Trauma | | | | | 1 | | | |
| 14 | M | 13 | Trauma | 1 | 1 | 1 | | 1 | | | |
| 16 | F | 13 | Trauma | | 1 | 1 | | | | | |
| 49 | M | 16 | Trauma | | 1 | 1 | | | | | |
| 4 | F | 18 | Other | | 1 | 1 | | | | | |
| 50 | F | 18 | Trauma | | 1 | 1 | | | | | |
| 39 | M | 20 | Trauma | | 1 | 1 | 1 | 1 | 1 | 1 | |
| **115** | M | 21 | Trauma | | | | | | | | 1 |
| 17 | M | 23 | Trauma | 1 | 1 | 1 | 1 | | | | |
| 92 | M | 24 | Anaphilatic schock | | | | | | | | 1 |
| 20 | M | 26 | Other | 1 | 1 | 1 | 1 | | | | |
| 112 | M | 27 | SVH | | | | | 1 | | | |
| **117** | F | 28 | Other | | | | | | | | 1 |
| 22 | M | 29 | Trauma | | 1 | 1 | 1 | | | | 1 |
| **116** | F | 29 | Trauma | | | | | | | | 1 |
| 5 | M | 30 | Trauma | 1 | 1 | 1 | 1 | 1 | | | |
| **120** | F | 34 | Asphyxia | | | | | | | | 1 |
| 10 | M | 37 | Trauma | 1 | 1 | 1 | 1 | | 1 | 1 | |
| 80 | M | 37 | Trauma | | | | | 1 | | | |
| **119** | M | 38 | Gunfire | | | | | | | | 1 |
| 6 | M | 43 | SVH | 1 | 1 | 1 | | | 1 | 1 | |
| 44 | M | 43 | SVH | | | | | 1 | | | |
| 7 | M | 44 | SVH | 1 | 1 | 1 | | 1 | | | |
| 45 | M | 45 | Trauma | | 1 | 1 | | | | | |
| 84 | M | 49 | Meningitis | | | | | 1 | | | |
| 13 | F | 50 | SVH | | 1 | 1 | | | | | |
| 15 | M | 50 | SVH | | 1 | 1 | | 1 | 1 | 1 | |
| 24 | M | 50 | SVH | | 1 | 1 | | 1 | 1 | | |
| 23 | F | 53 | SVH | | 1 | 1 | | | | | |
| 86 | F | 53 | SVH | | | | | | 1 | 1 | |
| 2 | M | 58 | SVH | | 1 | 1 | | | | | |
| 25 | F | 58 | SVH | | 1 | 1 | | | | | |
| 28 | M | 58 | Trauma | 1 | 1 | 1 | | | | | |
| 54 | M | 58 | SVH | | | | | 1 | | | |

| | | | | | | | | | | | |
|---|---|---|---|---|---|---|---|---|---|---|---|
| 1 | M | 59 | SVH | | 1 | 1 | | 1 | | | |
| 35 | F | 59 | SVH | | 1 | 1 | | | | | |
| 21 | F | 60 | SVH | | 1 | 1 | | | | | |
| 37 | F | 61 | Other | | 1 | 1 | | | | | |
| 11 | F | 63 | Trauma | | 1 | 1 | | | | | |
| 26 | M | 66 | SVH | 1 | 1 | 1 | | 1 | | | |
| 12 | F | 67 | SVH | | | 1 | | | | | |
| 27 | F | 67 | SVH | | 1 | | | | | | |
| 3 | F | 69 | SVH | | 1 | 1 | | | | | |
| 41 | F | 69 | SVH | | 1 | 1 | | | | | |
| 38 | F | 70 | post-anossic encephalopathy | | 1 | 1 | | | | | |
| 51 | M | 70 | SVH | | | | | 1 | 1 | 1 | |
| 110 | M | 70 | SVH | | | | | 1 | | | |
| 78 | M | 71 | SVH | | | | | 1 | | | |
| 47 | M | 72 | SVH | | 1 | 1 | | 1 | | | |
| 8 | F | 73 | SVH | | 1 | 1 | | | | | |
| 99 | F | 73 | SVH | | | | | | | 1 | |
| 113 | M | 73 | Trauma | | | | | 1 | | | |
| 40 | M | 74 | SVH | | 1 | 1 | | | | | 1 |
| 43 | F | 74 | SVH | | | | | | 1 | 1 | |
| 52 | M | 74 | SVH | | | | | 1 | | | |
| 56 | M | 74 | SVH | | | | | | | | 1 |
| 46 | M | 75 | SVH | | 1 | 1 | | | 1 | 1 | 1 |
| 90 | M | 75 | Trauma | | | | | 1 | | | 1 |
| 9 | F | 76 | SVH | | 1 | 1 | | | | | |
| 30 | M | 76 | SVH | 1 | 1 | 1 | 1 | | | | |
| 32 | F | 77 | SVH | | 1 | 1 | | | | | |
| 42 | M | 77 | SVH | | 1 | 1 | 1 | | 1 | 1 | |
| 48 | F | 78 | SVH | | 1 | 1 | | | | | |
| 89 | M | 81 | SVH | | | | | 1 | | | |
| 33 | M | 82 | SVH | | 1 | 1 | 1 | 1 | | | |
| 36 | M | 82 | Trauma | | 1 | 1 | 1 | 1 | | | |
| 29 | M | 83 | SVH | 1 | 1 | 1 | 1 | 1 | | | |
| 114 | M | 83 | Trauma | | | | | | | | 1 |
| 19 | M | 87 | SVH | 1 | 1 | 1 | 1 | 1 | 1 | 1 | 1 |
| 34 | F | 90 | SVH | | 1 | 1 | | | | | |
| **118** | M | **92** | Trauma | | | | | | | | 1 |

| | | | | | | | | | | | |
|---|---|---|---|---|---|---|---|---|---|---|---|
| **TOTAL** | | | **71** | **12** | **45** | **45** | **12** | **26** | **11** | **11** | **14** |

SVH is Spontaneous vascular hemorrhage

Pink colour is referred to Female gender
