# Supplementary material for: Identification of miR‐31‐5p, miR‐141‐3p, miR‐200c‐3p, and GLT1 as human liver aging markers sensitive to donor–recipient age‐mismatch in transplants
Source: Aging Cell. 2016 Dec 20;16(2):262–72. doi: 10.1111/acel.12549 (PMC5334540; doi:10.1111/acel.12549)
Supplement: Supplementary file 15 [file ACEL-16-262-s015.pdf]

Blood samples											
ID	Age	Liver disease	anti-HCV	HBsAg	Donor age (years)	Δ AGE (Donor-Recipient)	Age-mismatch Groups	Pre-transplant blood	Post-transplant blood	Immunosuppressive drug therapy (induction)	TBIL (mg/dL)
1	62	HCC with HCV-cirrhosis	POS	NEG	59	-3	B		N-Glycomics	Tacrolimus, Steroids	3.3
3	51	Cryptogenic cirrhosis	NEG	NEG	69	18	C		N-Glycomics	Tacrolimus, Steroids	9.2
6	57	Alcoholic cirrhosis	NEG	NEG	43	-14	A		N-Glycomics	Tacrolimus, Steroids	3.7
9	42	Alcoholic cirrhosis	NEG	NEG	76	34	C		N-Glycomics	Tacrolimus, Steroids	12.0
10	52	HCV-cirrhosis	POS	NEG	37	-15	A		N-Glycomics	Tacrolimus, Mycophenolate , Steroids	0.6
17	46	HCC with HCV and alcoholic cirrhosis	POS	NEG	23	-23	A		N-Glycomics	Tacrolimus, Steroids	12.2
19	60	Alcoholic liver cirrhosis with HCC	NEG	NEG	87	27	C		N-Glycomics	Tacrolimus, Steroids	3.4
22	36	HCC with HBV and HDV-cirrhosis	NEG	POS	29	-7	B		N-Glycomics	Tacrolimus, Basiliximab, Steroids	1.1
22	56	Polycystic liver and kidney disease	NEG	NEG	29	-27	A		N-Glycomics	Tacrolimus, Alemtuzumab	0.3
24	66	HCC with HCV-cirrhosis	POS	NEG	50	-16	A		N-Glycomics	Tacrolimus, Steroids	16.8
27	64	HCV-cirrhosis	POS	NEG	67	3	B		N-Glycomics	Tacrolimus, Basiliximab, Steroids	4.2
34	44	HCC with HBV and HDV-cirrhosis	NEG	POS	90	46	C		N-Glycomics	Tacrolimus, Steroids	0.9
39	49	Amyloidosis	NEG	NEG	20	-29	A		N-Glycomics	Thymoglobulin, Cyclosporine, Mycophenolate, Steroids	2.0
40	66	HCC with HCV-cirrhosis	POS	NEG	74	8	B		N-Glycomics	Everolimus, Steroids	43.1
41	64	HCC with HCV-cirrhosis	POS	NEG	69	5	B		N-Glycomics	Tacrolimus, basiliximab, Steroids	0.7
43	56	Alcoholic cirrhosis	NEG	NEG	74	18	C		N-Glycomics	Tacrolimus, Steroids	3.9
45	45	HCV-cirrhosis	POS	NEG	45	0	B		N-Glycomics	Tacrolimus, Basiliximab, Steroids	10.4
46	57	Alcoholic liver cirrhosis with HCC	NEG	NEG	75	18	C		N-Glycomics	Tacrolimus, Steroids	2.7
48	25	Primary sclerosing cholangitis	NEG	NEG	78	53	C		N-Glycomics	Tacrolimus, Sirolimus, Steroids	0.5
51	26	HBV and HDV-cirrhosis	NEG	POS	70	44	C	N-Glycomics	N-Glycomics	Tacrolimus, Steroids	1.4
54	46	HBV and HDV-cirrhosis	NEG	POS	58	12	C	N-Glycomics	N-Glycomics	Tacrolimus, Steroids	35.0

55	50	HCV-cirrhosis	POS	NEG	68	18	C	N-Glycomics	N-Glycomics	Tacrolimus, Basiliximab, Steroids	28.0
56	50	Alcoholic cirrhosis	NEG	NEG	74	24	C	N-Glycomics	N-Glycomics	Tacrolimus, Steroids	3.7
78	40	HCV-cirrhosis	POS	NEG	71	31	C	N-Glycomics	N-Glycomics	Tacrolimus, Basiliximab, Steroids	2.3
79	51	HBV-cirrhosis	NEG	POS	54	3	B	N-Glycomics	N-Glycomics	Tacrolimus, Steroids	1.6
81	55	HCC with HBV and HDV-cirrhosis	NEG	POS	89	34	C	N-Glycomics	N-Glycomics	Tacrolimus, Steroids	0.4
84	49	HCC with HBV-cirrhosis	NEG	POS	49	0	B	N-Glycomics	N-Glycomics	Tacrolimus, Steroids	4.7
86	69	HCC with HCV-cirrhosis	POS	NEG	53	-16	A	N-Glycomics	N-Glycomics	Tacrolimus, Basiliximab, Steroids	0.9
88	45	HCC with HCV-cirrhosis	POS	NEG	12	-33	A	N-Glycomics	N-Glycomics	Tacrolimus, Basiliximab, Steroids	3.1
91	34	Budd-Chiari syndrome	NEG	NEG	87	53	C	N-Glycomics	N-Glycomics	Tacrolimus, Steroids	1.3
99	48	HCC with HCV-cirrhosis	POS	NEG	73	25	C	N-Glycomics	N-Glycomics	Tacrolimus , basiliximab, Steroids	2.3
104	54	HBV and HDV-cirrhosis	NEG	POS	88	34	C		N-Glycomics	Tacrolimus, Steroids	12.6
107	52	Polycystic liver and kidney disease	NEG	NEG	64	12	C		N-Glycomics	Tacrolimus, Steroids	0.3
109	58	HCV-cirrhosis	POS	NEG	65	7	B		N-Glycomics	Tacrolimus, Steroids	1.7
114	46	HBV and HDV-cirrhosis	NEG	POS	83	37	C	N-Glycomics	N-Glycomics	Tacrolimus, Steroids	28.2
121	54	Primary biliary cirrhosis	NEG	NEG	12	-42	A	N-Glycomics	N-Glycomics	Tacrolimus, Steroids	7.9

Pre-Transplant biochemistry								Post-transplant biochemistry						
IBIL (mg/dL)	ALB (g/100mL)	AST (IU/L)	ALT (IU/L)	GGT (IU/L)	ALP (IU/L)	Months post transplant	Immunosuppressive drug therapy (maintenance)	TBIL (mg/dL)	IBIL (mg/dL)	ALB (g/100mL)	AST (IU/L)	ALT (IU/L)	GGT (IU/L)	ALP (IU/L)
2.3	4.0	43.0	36.0	15.0	224.0	20	Tacrolimus , Steroids	1.5	0.6	3.8	88.0	94.0	149.0	287.0
4.5	3.1	42.0	19.0			8	Tacrolimus , Steroids	1.3	0.9	4.0	25.0	34.0	134.0	197.0
2.6	4.7	39.0	30.0	15.0	69.0	23	Tacrolimus , Mycophenolate, Steroids	0.9	0.7	4.5	15.0	17.0	59.0	269.0
4.0	3.4	64.0	97.0			19	Tacrolimus , Mycophenolate	0.3	0.2		25.0	26.0	29.0	168.0
0.4	3.0	54.0	63.0			19	Tacrolimus , Mycophenolate	0.5	0.2	4.0	101.0	114.0	176.0	265.0
4.0	3.4	92.0	49.0			22	Sirolimus, Steroids	0.5	0.2		242.0	181.0	411.0	504.0
2.5	3.8	25.0	17.0			20	Tacrolimus	0.5	0.1	3.9	32.0	16.0	13.0	175.0
0.6	4.8	44.0	72.0			17	Everolimus	0.6	0.4	4.5	18.0	21.0	27.0	254.0
0.2	4.7	21.0	8.0			17	Tacrolimus	0.7	0.4	4.0	17.0	12.0	13.0	174.0
5.1	3.2	390.0	398.0	37.0	152.0	18	Everolimus , Steroids	0.5	0.4	4.5	22.0	36.0	116.0	408.0
2.0	3.0	28.0	15.0	15.0	263.0	19	Tacrolimus , Steroids	0.5	0.3		135.0	189.0	24.0	257.0
0.4	4.3	45.0	59.0	64.0	138.0	1	Tacrolimus , Steroids	1.2	0.4	4.6	26.0	31.0	89.0	143.0
0.9	5.0	19.0	25.0			8	Tacrolimus , Steroids	0.7	0.5	3.9	21.0	16.0	8.0	215.0
12.4	4.2	47.0	17.0			11	Tacrolimus , Steroids	2.4	1.6	3.9	50.0	61.0	69.0	197.0
0.4	4.1	31.0	27.0	21.0	221.0	22	Everolimus, Steroids	1.0	0.6	4.3	29.0	37.0	55.0	313.0
1.9	3.4	23.0	9.0			22	Tacrolimus	0.8	0.4		16.0	14.0	20.0	249.0
4.6	4.5	75.0	46.0			26	Cyclosporine , Steroids	0.8	0.6	4.0	23.0	19.0	46.0	199.0
1.6	4.3	45.0	30.0	112.0	267.0	10	Tacrolimus , Steroids	0.8	0.4	4.7	23.0	31.0	47.0	149.0
0.3	4.6	63.0	79.0			8	Tacrolimus, Sirolimus , Steroids	1.4	0.8	4.1	35.0	38.0	87.0	149.0
0.8	3.3	78.0	60.0			7	Tacrolimus	0.3	0.2	3.9	17.0	18.0	15.0	156.0
5.0	4.5	107.0	62.0			12	Tacrolimus , Sirolimus , Steroids	0.6	0.5	3.9	48.0	42.0	118.0	316.0

3.5	3.0	120.0	36.0	181.0	294.0	5	Cyclosporine , Steroids	2.7	1.8	2.7	104.0	95.0	554.0	347.0
2.2	3.8	36.0	16.0			13	Tacrolimus , Sirolimus , Steroids	0.3	0.2		21.0	19.0	19.0	310.0
0.4	3.2	141.0	92.0	160.0	360.0	8	Tacrolimus , Steroids	1.9	0.5	4.0	88.0	64.0		
0.7	3.3	46.0	47.0			6	Tacrolimus , Sirolimus , Steroids	0.9	0.5		45.0	52.0	291.0	355.0
0.3	4.3	26.0	7.0			18	Sirolimus	0.3	0.2		20.0	11.0	47.0	70.0
1.2		139.0	84.0			7	Tacrolimus , Steroids	0.4	0.3		20.0	43.0	48.0	210.0
0.4	3.8	39.0	39.0	36.0	213.0	6	Tacrolimus, Steroids	1.2	0.6		43.0	90.0	204.0	185.0
1.0	2.4	80.0	28.0			4	Tacrolimus, Steroids	0.3	0.2		27.0	25.0	156.0	185.0
0.5	3.8	1352.0	2176.0	57.0	301.0	26	Tacrolimus , Mycophenolate	0.4	0.2		21.0	17.0	13.0	54.0
0.9	3.4	173.0	174.0			7	Tacrolimus, Steroids	0.9	0.5		177.0	187.0	251.0	386.0
6.3	3.0	66.0	25.0			25	Tacrolimus	1.2	0.7		15.0	8.0	9.0	116.0
0.1	4.5	8.0	12.0	46.0	183.0	23	Tacrolimus, Steroids	0.6	0.4		15.0	20.0	15.0	85.0
0.8	3.6	49.0	33.0			9	Cyclosporine , Steroids	1.1	0.3	4.1	55.0	61.0	45.0	164.0
4.7	3.8	112.0	46.0	20.0	161.0	20	Tacrolimus	0.4	0.3		17.0	15.0	42.0	129.0
2.1	3.4	135.0	134.0			14	Tacrolimus, Steroids	0.5	0.3		18.0	17.0	8.0	77.0
